# Redescription of two subterranean amphipods *Niphargus molnari* Méhely, 1927 and *Niphargus gebhardti* Schellenberg, 1934 (Amphipoda, Niphargidae) and their phylogenetic position

**DOI:** 10.3897/zookeys.509.9820

**Published:** 2015-06-24

**Authors:** Dorottya Angyal, Gergely Balázs, Valerija Zakšek, Virág Krízsik, Cene Fišer

**Affiliations:** 1 Department of Zoology, Hungarian Natural History Museum, Baross u. 13, 1088 Budapest, Hungary; 2 Doctoral School of Animal-and Agricultural Environmental Sciences, Department of Animal Sciences and Animal Husbandry, Georgikon Faculty, University of Pannonia, Deák Ferenc u. 16, 8360 Keszthely, Hungary; 3 Department of Systematic Zoology and Ecology, Eötvös Loránd University, Pázmány Péter sétány 1/C, 1117 Budapest, Hungary; 4 Department of Biology, Biotechnical Faculty, University of Ljubljana, Jamnikarjeva 101, SI-1000 Ljubljana, Slovenia; 5 Laboratory of Molecular Taxonomy, Hungarian Natural History Museum, Ludovika tér 2, 1083 Budapest, Hungary

**Keywords:** Hungary, Mecsek Mts., *Niphargus*, redescription, morphology, phylogeny, endemism, SEM

## Abstract

A detailed redescription of two endemic, cave-dwelling niphargid species of the Hungarian Mecsek Mts., *Niphargus molnari* Méhely, 1927 and *Niphargus gebhardti* Schellenberg, 1934 is given based on newly collected material. Morphology was studied under light microscopy and with scanning electon microscopy. Morphological descriptions are complemented with mitochondrial cytochrome c oxidase subunit I (COI) sequences as barcodes for both species and with notes on their ecology. Using three independent molecular markers we showed that *Niphargus gebhardti* belongs to the clade distributed between Central and Eastern Europe, whereas phylogenetic relationship of *Niphargus molnari* to the rest of *Niphargus* species is not clear. The two species from the Mecsek Mts. are phylogenetically not closely related. Both species need to be treated as vulnerable according to IUCN Red List of Threatened Species.

## Introduction

**Figure 1.**
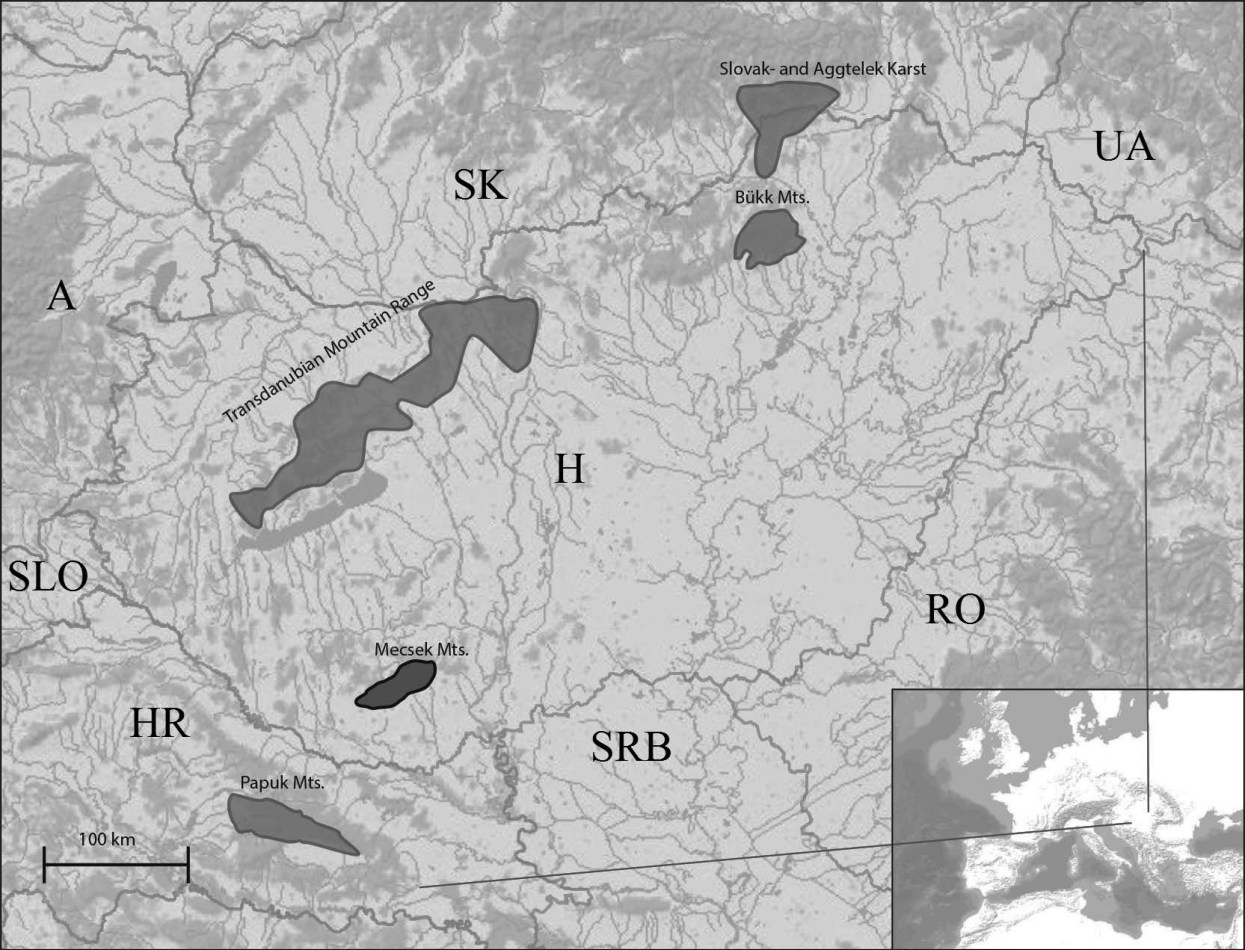
Location of the Mecsek Mts. and the nearby isolated mountain ranges within Europe.

Fragmented mountain areas in East-Central Europe had been suggested to be centres of endemisms that evolved through a complex geological history including Eocene marine regression-transgression cycles and Pleistocene glacial cycles (Hou et al. 2013, Meleg et al. 2013, Mamos et al. 2014). The Mecsek is one of these isolated mountain ranges, that is situated in Southern Hungary and surrounded by Pannonian plains. The closest mountain ranges are the Croatian Papuk Mts. (80 km) and the Hungarian Transdanubian Mts. (150 km) (Fig. 1). The area is small of approximately 545 km². In biological sense, it is populated by numerous endemic species the origin of which may date back to Tertiary and which therefore apparently have survived mass extinctions in glacial periods. The upper geological layers comprise of Triassic and Jurassic limestones and dolomites, where extensive karstification has created over 200 caves. The subterranean environment of the area harbours numerous terrestrial and aquatic highly endemic invertebrates, known only from one or a few caves. Although the region apparently harbours an important piece of European and Hungarian natural heritage, until now only one species, the Hungarian blind snail (*Bythiospeum hungaricum* (Soós, 1927)) has been protected by law. A serious impediment for conservation biology is that our knowledge of species is only limited, beginning with poor taxonomic descriptions. The aim of this study is to bridge this gap at the most basic level. We morphologically redescribe and present phylogenetic relationships of two amphipod species from the genus *Niphargus*, both endemic to this area.

*Niphargus molnari* Méhely, 1927 was described from the stream of the Mánfai-kőlyuk Cave (Méhely 1927). The description is not detailed, as it contains only a few information about the body lenght, the pereonits, the pleon segments, the first antenna, the uropods and the telson, and two drawings about the epimeral plates and the pereion segments. Further drawing of the right lacinia mobilis can be found in Méhely’s summarizing work (Méhely 1941). At approximately the same period the species was also studied by Schellenberg, who analysed samples fom Abaligeti Cave. In his early study he first treated it as *Niphargus leopoliensis molnari* (Schellenberg, 1933), but later he acknowledged its species status and supplemented description with data about the seta number of the palpus of the first maxilla (Schellenberg 1935). The species was found in the Mánfai-kőlyuk Cave (Gebhardt 1933, 1934, 1963, 1967) and in the stream of the Abaligeti Cave too (Gebhardt 1934, 1963, 1967). Recently, the species was found in other two localities, the Spirál Sinkhole and the Vadetetős Sinkhole (Angyal and Balázs 2013). During our research in the caves of the Western Mecsek between 2010 and 2013, the species could not have been re-collected on the type locality, which is supposedly related to the artificial utilization of the Mánfai-kőlyuk Cave. The intrusive introduction of waterworks in the 1960-s and 1970-s has caused irreversible changes in the cave’s character, hidrology and ecosystem (Angyal 2012).

*Niphargus gebhardti* Schellenberg, 1934 was described from the pools formed by dripping water of the Abaligeti Cave, originally as *Niphargus foreli gebhardti* (Schellenberg 1934). Brief description reports on only few characters, like the pereopods, the antennae and the mouth parts, and two drawings about the second gnathopod’s propodus and the telson. Later the author gave additional data on the body length and the telson (Schellenberg 1935). Gebhardt mentioned the species’distribution from pools of the Abaligeti Cave’s main passage in various papers (Gebhardt 1934, 1963, 1967). The species rank was proposed for the first time in Méhely’s synthetic work (Méhely 1941), wherein a drawing of the pleopod’s retinacles and some data about the lacinia mobilis are also presented. Dudich (1941) discussed ‘*Niphargus foreli gebhardti*’ from the Abaligeti Cave as a fauna element of the historical Hungary. More recent sampling revealed new records of the species from Vadetetős Sinkhole, Szajha-felső Sinkhole, Spirál Sinkhole, Gilisztás Cave and Trió Cave (all Mecsek Mts.; see Angyal and Balázs 2013).

The holotypes of both species are either in an unknown place or had been destroyed. Although we identified the distinguishing characters of *Niphargus gebhardti* and *Niphargus molnari*, and presented comparative drawings of them (Angyal and Balázs 2013), the morphology of both species is unsuficiently known and cannot be used in a broader comparative research of *Niphargus*. In order to follow modern trends in taxonomy, we revised all possible sources of data that might increase the robustness of taxonomic conclusions (Padial et al. 2010). We provide a detailed and richly illustrated redesription of *Niphargus molnari* and *Niphargus gebhardti* with cytochrome c oxidase subunit I (COI) sequences as barcodes. We also present comparative scanning electron micrographies which are – to our knowledge – the first comparative micrographies of *Niphargus*. Moreover, we present phylogenetic relationships of both species within the genus *Niphargus* using three independent molecular markers and summarize field observations that may indicate species’ ecology.

## Material and methods

### Sampling sites and sampling

**Figure 2.**
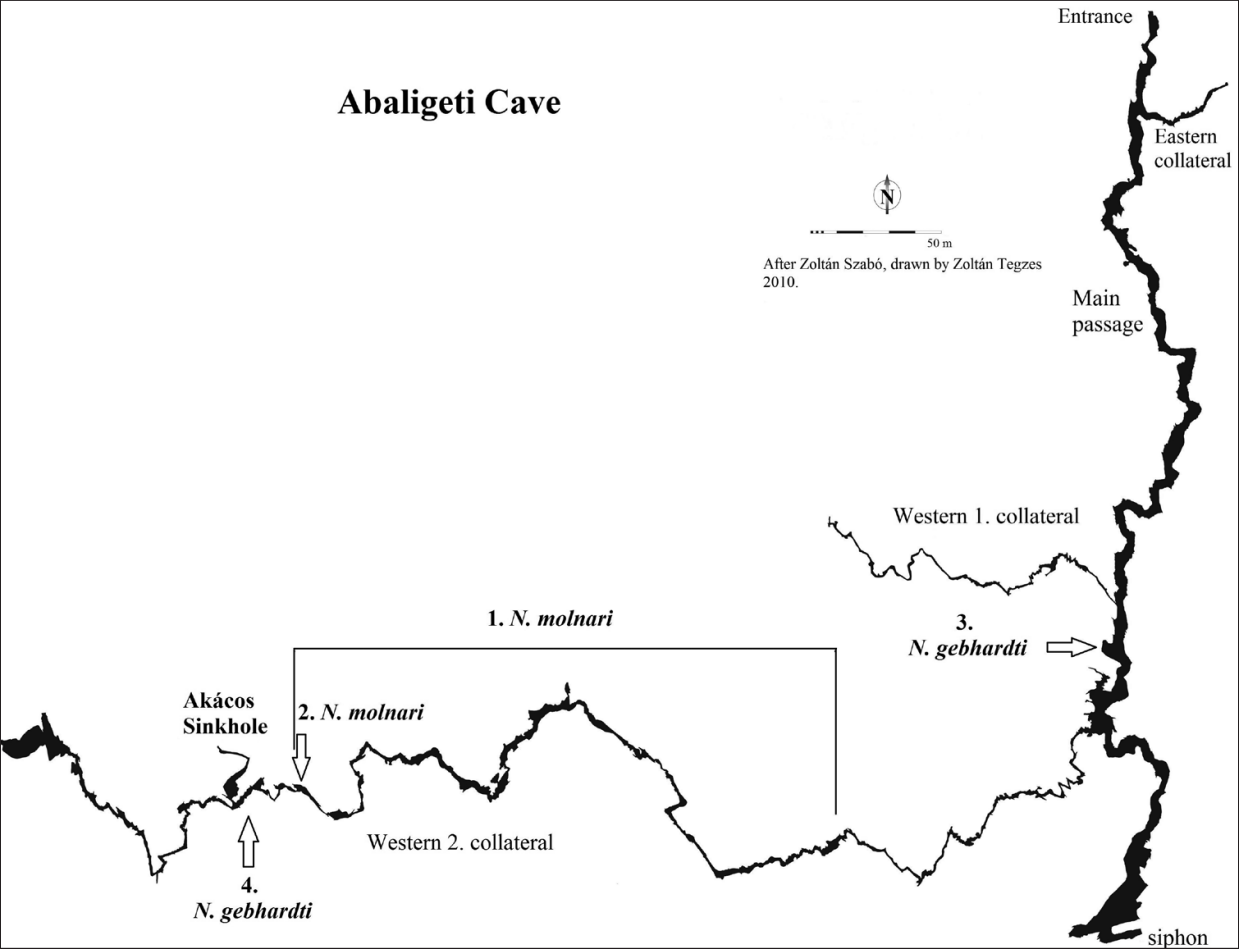
Distribution of the two species within the Abaligeti Cave. **1** *Niphargus molnari* along the stream of the Western 2. collateral **2** *Niphargus gebhardti* in a permanent pool of ’Karthago romjai’ **3** *Niphargus gebhardti* in a permanent pool near the Akácos Sinkhole’s entrance.

Samples for the redescription were collected in the Abaligeti Cave (N46°8'11.89", E18°6'59.40"), which is located in Southern Hungary, Western Mecsek in Abaliget village, near Pécs city. The altitude of the cave entrance is 219 m above sea level. With its three collaterals and the main passage, the total length of the cave is 2000 m. Its lowest point below the entrance is 10 m, while its highest point is 38 m. Shallow pools of water in the cave are of two types: some are formated by dripping water of the dripstones whereas others are filled during floods and contain residual water. The cave was regulary visited between 2010 and 2013 to characterize its fauna. For the morphological and molecular taxonomic analysis in total 18 and 20 specimens of *Niphargus molnari* and *Niphargus gebhardti* respectively were collected on 23 March 2013. *Niphargus molnari* was found in the stream of the Western 2. collateral and *Niphargus gebhardti* was collected from a permanent pool in a lateral chamber of ‘Karthago romjai’ hall in the main passage and from a pool at the end of Western 2. collateral, near Akácos Sinkhole’s entrance (Fig. 2). An additional specimen of *Niphargus gebhardti* for molecular studies was collected from a pool of the Szajha-felső Sinkhole (46°8'5.4"N, 18°7'8.22 E) 30 m vertical distance and 100 m horizontal distance from the entrance. The cave is situated in the area of a platform right above the Abaligeti Cave, 283 m above sea level. The two caves are supposedly connected, their entrances are approximately 1 km from each other (Dezső 2011). Specimens were collected using entomological (soft) forceps and were fixed and stored in 96% ethanol.

### Morphological studies

Cleared and stained exoskeletons of 10 (*Niphargus molnari*) and 11 (*Niphargus gebhardti*) specimens were dissected under a Leica MZ75 and a Leica M125 stereomicroscope. Slides were examined using a Leica DM 1000 light microscope. Drawings were made using a drawing tube mounted on the light microscope. Measurements were made using the AnalySIS Program Package, the computer was connected with a Zeiss Axioscope II light microscope. In total 230 morphological characters on each speciemens were examined according to the characters of the DELTA program package (Fišer et al. 2009) which were recorded in an Excel data matrix. Scanning micrographs of two individuals of each species about the main characters were made with a HITACHI S-2600 N scanning electron microscope. Specimens were placed in absolute alcohol for one day, then cleaned in an EMAG Emmi-16 Ultrasonic Cleaner and dried out on air. Dry samples were sticked onto holders and were sputter-coated by gold-palladium. Micrographs were digitally edited.

### Molecular studies

DNA extraction was performed using QIAamp DNA Microcit® (Qiagen) or Sigma Aldrich GenElute Mammalian Genomic DNA Miniprep Kit® following the manufacturer’s instructions. Only a few pereopods were used for DNA isolation of each animal. The following primer pairs were used for PCR amplifications of COI, 28S rDNA fragment and histone (H3). For COI: LCO 1490 – HCO 2198 (Folmer et al. 1994), for 28S rDNA: 28S lev2 – 28S des2 or 28S rtest2 (Verovnik et al. 2005, Zakšek et al. 2007) and H3aF2–H3aR2 (Colgan et al. 2000) for histone (H3). Details on PCR conditions are listed in Suppl. material 1. PCR products were cleaned using Roche High Pure Purification Kit® or Exonuclease I and Alkaline Phosphatase (Fermentas, Germany) according to manufacturer’s instruction. The fragments were sequenced in both directions using PCR amplification primers using ABI 3130 sequencer in the Laboratory of Molecular Taxonomy in Budapest or Macrogen Europe (Amsterdam, The Netherlands). Contigs were assembled and sequences were edited using Geneious Pro 5.5.6. (Biomatters, New Zeland).

### Phylogenetic analysis

In order to recover phylogenetic relationships of *Niphargus molnari* and *Niphargus gebhardti* within the genus *Niphargus*, a dataset of three molecular markers were complied, using available *Niphargus* sequences from previous studies (see Suppl. material 2 for references) and *Synurella ambulans* as outgroup taxon (Švara et al. submitted, Meleg et al. 2013). Altogether 104 taxa were included in the final dataset. List of taxa and sequences with GenBank accession numbers used in the analyses are listed in Suppl. material 2. The sequences were aligned using MAFFT 7 (Katoh and Standley 2013). Each sequence alignment was concatenated to the joint dataset and analysed as a single dataset in phylogenetic analysis. The length of combined dataset, including sequences of COI, 28S rDNA and H3 was 2068bp. A general time-reversible model with a proportion of invariant sites and a gamma distribution of rate heterogeneity (GTR+I+Γ) assuming six discrete gamma categories was chosen as the most appropriate model according to AIC and BIC criteria, using ModelGenerator (Keane et al. 2006). Phylogenetic relationships were reconstructed with Bayesian inference (BA) using MrBayes v3.2 (Ronquist and Huelsenbeck 2003). Two parallel searches with four chains each were run for 20 million generations, sampled every 1000^th^ generation. After discarding the ﬁrst 25% of the sampled trees, the ﬁnal tree was constructed according to the 50% majority rule. MrBayes phylogenetic analysis was run on the CIPRES Science Gateway, www.phylo.org (Miller et al. 2012).

## Results

### Redescription of *Niphargus molnari* Méhely, 1927 Order Amphipoda Latreille, 1816 Suborder Gammaridea Latreille, 1802 Family Niphargidae G. Karaman, 1962 Genus Niphargus Schiödte, 1849

#### Niphargus molnari

Taxon classificationAnimaliaAmphipodaNiphargidae

Méhely, 1927

Niphargus molnari sp. n.: Méhely 1927 type locality: Mánfai-kőlyuk Cave; Data from the original description is available in Suppl. material 3.
Niphargus leopoliensis molnari : Schellenberg 1933, samples from the Abaligeti Cave, morphological data.
Niphargus molnari : Schellenberg 1935, morphological data.
Niphargus leopoliensis molnari , *Niphargus molnari*: Gebhardt 1933, 1934, 1963, 1967 distributional data
Niphargus molnari : Méhely 1941 additional morphological data.
Niphargus molnari : Angyal and Balázs 2013 morphological and distributional data.

##### Material examined for redescription.

7 females and 3 males from the stream of the Western 2. collateral of the Abaligeti Cave (Cadastre number: 4120-1, Hungarian Cave Cadastre), collected in 23 March 2013 (leg. D. Angyal and A. Illés), dissected and mounted on slides; additional 4 specimens not dissected. Slides were deposited in the Collection of Crustaceans of the Hungarian Natural History Museum with the following codes: N.MOL-02, N.MOL-03, N.MOL-04, N.MOL-06, N.MOL-07, N.MOL-08, N.MOL-09, N.MOL-10, N.MOL-11, N.MOL-12. Diagnostic voucher number of specimen used for molecular studies: NB555 (*Niphargus molnari*, coll. data: Abaligeti Cave, Western 2. collateral, stream, 23 March 2013, leg. D. Angyal & A. Illés).

COI Gen Bank Accession Number: KP967552

##### Diagnosis.

Small to medium-sized niphargid; epimeral plate III postero-ventral corner sharply inclined. Telson with 3–4 apical spines, 1–3 lateral spines, 0–2 lateral plumose setae, 0–2 spines in cleft, dorsal surface with 1–3 spines in mediobasal position. Maxilla I outer lobe with 7 spines, 1.-3. pluri-toothed, 4.-7. variable (uni-, bi-, pluri-toothed). Gnathopod I and gnathopod II dactyli with single seta on outer margin. Gills II-VI ovoid, approximately same size as pereopod VI coxa, posterior margin slightly concave. Pleopods I-III with 2 retinacles on each. Uropod I lenght of endopodite: length of exopodite ratio as 1.00: (1.00–1.20) on males and 1.00: (1.15–1.18) on females. Uropod III sexually dimorphic, exopodite rod-shaped, distal article of exopodite on males 83–115% of proximal article length and 18–73% on females.

##### Description.

**Figure 3.**
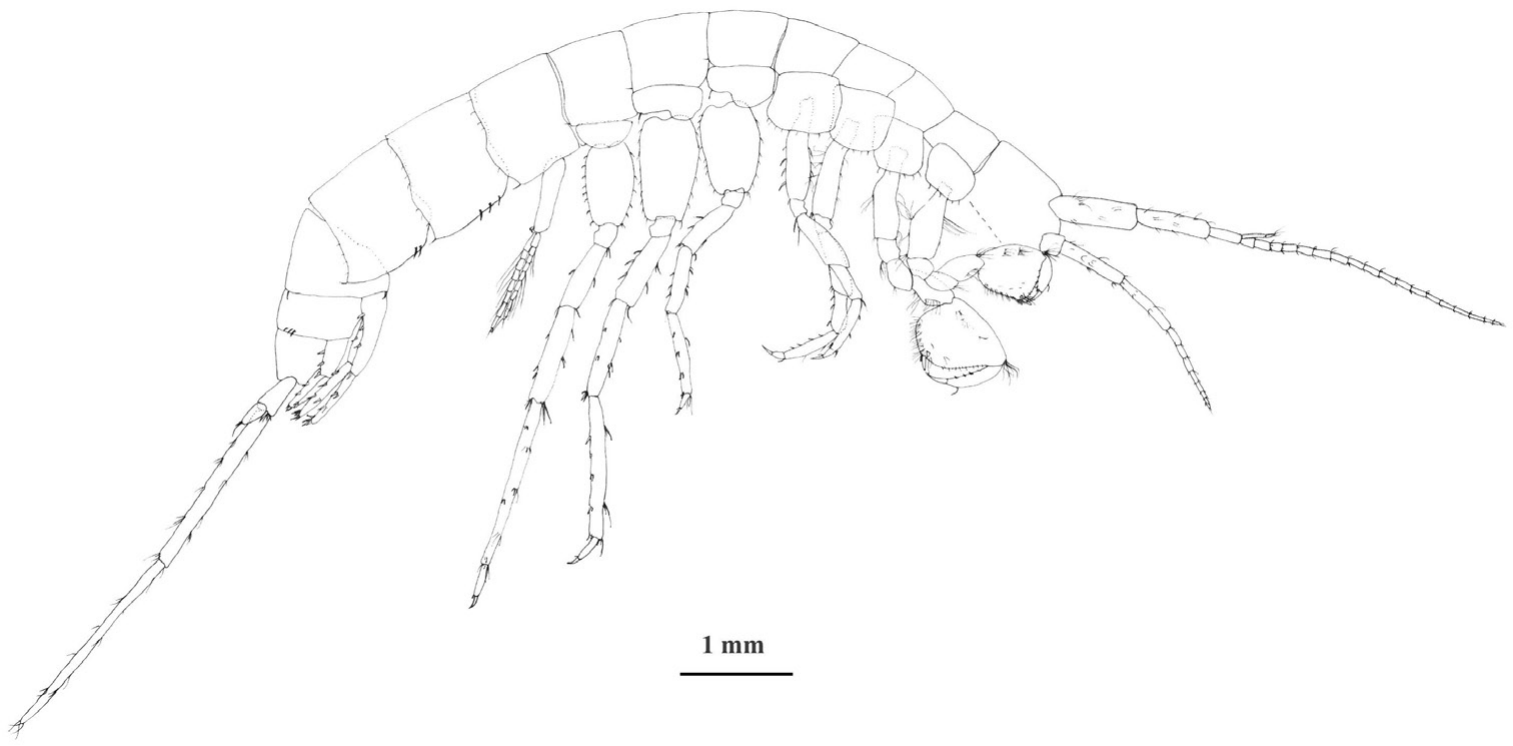
*Niphargus molnari*, male from the Abaligeti Cave, lateral view. Telson, mouthparts and pleopods II-III are not drawn.

**Figure 4.**
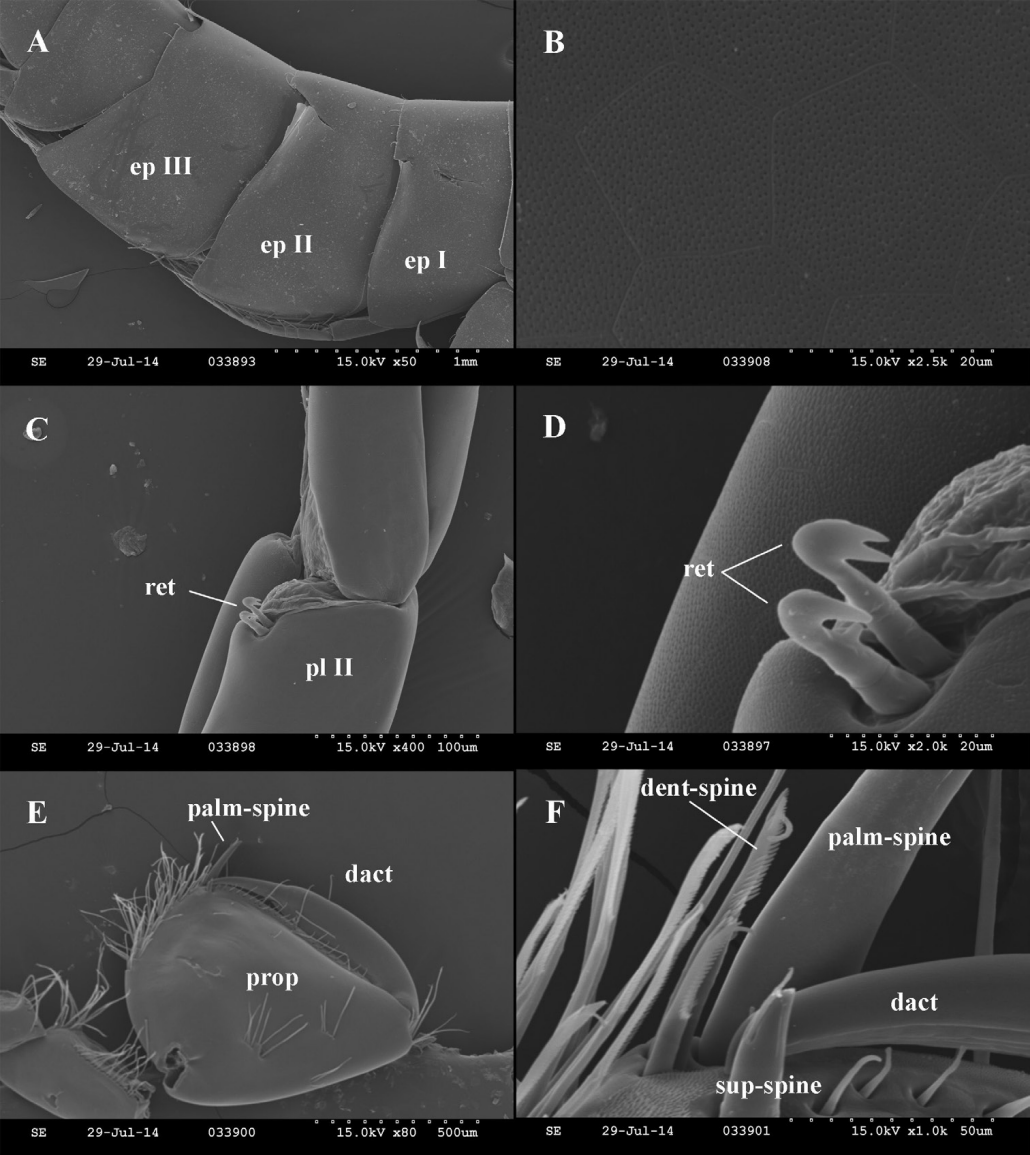
*Niphargus molnari*, scanning electron micrographs. **A** epimeral plates (Ep1-3 = epimeral plates 1-3) **B** honeybee-cell pattern on the exosceleton (tipical feature of amphipods) **C** pleopod with two retinacles (pl-r = pleopod ramus, ret = retinaculum) **D** retinaculi on the pleopod (ret = retinaculum) **E** gnathopod II propodus (prop = propodus, sup-spine = supporting spine, dact = dactylus) **F** palmar region of gnathopod II propodus (dent-spine = denticulated spine, sup-spine = supporting spine, n = nail, palm-spine = palmar spine).

Body and telson. Small to medium-sized species, females are 6.4 mm to 9.0 mm, males are 7.8 mm to 10.6 mm. Head length up to 13% of body length; rostrum absent. Pereonites I–VI without setae; pereonite V, VI, VII with 1 postero-ventral seta each. Pleonites I–III with 3–6 setae along dorso-posterior margin (Fig. 3). Epimeral plate II ventral and posterior margins straight or sinusoid, ventro-postero-distal corner approximately perpendicular and pointed; along ventral margin 1–3 spiniform setae; along posterior margin 4–6 thin setae (Figs 3, 4). Epimeral plate III ventral margin convex and posterior margin straight, ventropostero-distal corner sharply inclined, strongly produced; along ventral margin 2–3 spiniform setae; along posterior margin 4–6 thin setae (Figs 3, 4). Urosomite I postero-dorso-laterally with 1–2 spiniform seta; urosomite II postero-dorso-laterally with 2–3 spiniform setae; urosomite III without setae. Near insertion of uropod I 1 spiniform seta (Fig. 3).

Telson length: width as 1.0: 0.6–0.8; cleft 71–87% of length; lobes apically rounded. Telson spines (per lobe): 3–4 apical spines; lateral margins with 1–3 spine, 0–2 plumose setae; 0–2 in cleft spines, dorsal surface with 1–3 basal spines in mediobasal position (Figs 4, 9). Length of apical spines 20–25% of telson length.

**Figure 5.**
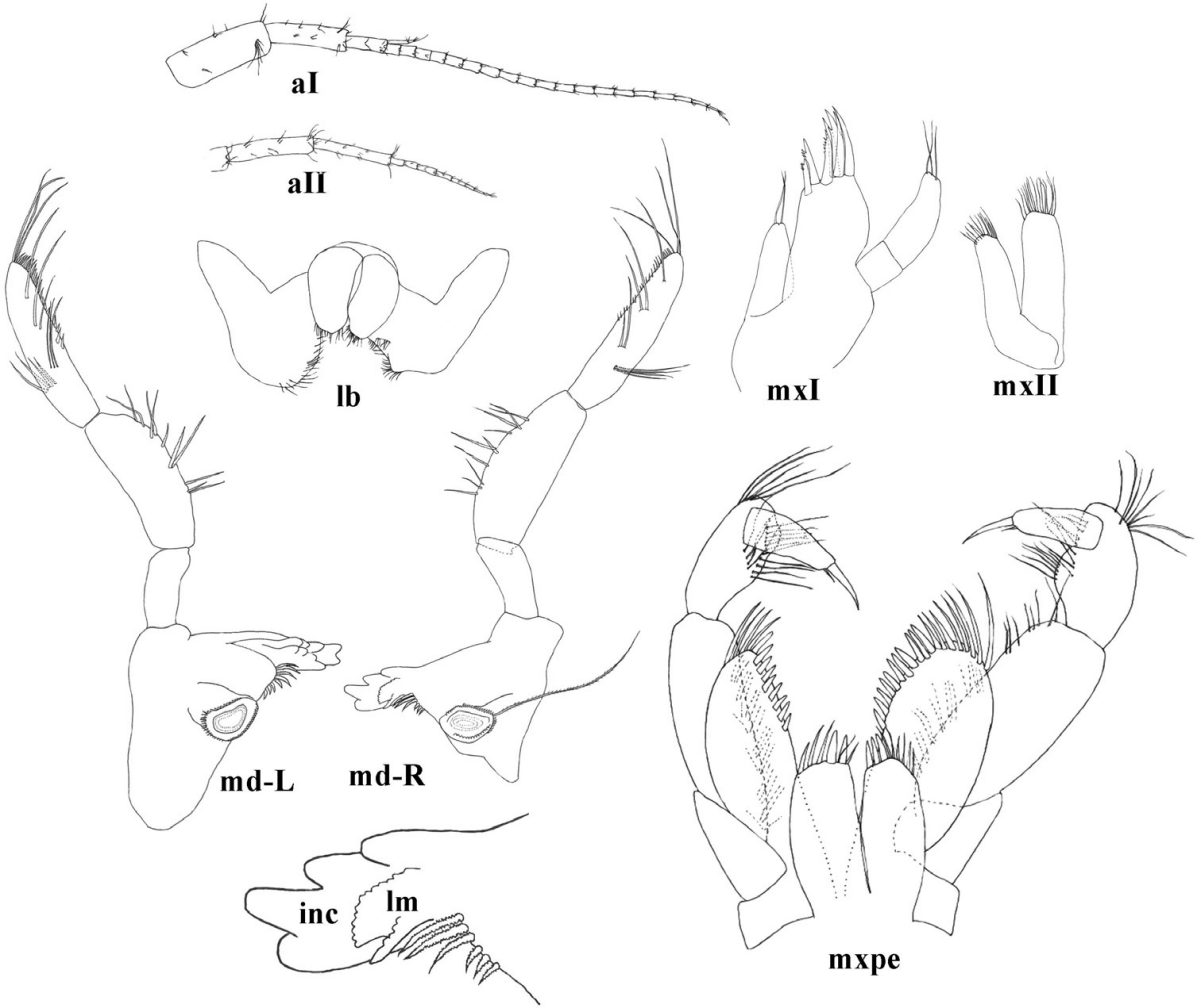
*Niphargus molnari*, aI = antenna I, aII = antenna II, mxI = maxillaI, mxII = maxilla II, md-R = right mandibula, lm = lacinia mobilis, inc = incisor, md-L = left mandibula, lb = labium, mxpe: maxilliped.

Antennae and mouthparts. Antenna I 35–48% of body length. Flagellum with up to 19 articles; each article with 1 long aesthetasc. Peduncle article 1: 2: 3 proportions 1.0: 0,78 (0.72–0.88): 0,4 (0.36–0.46). Proximal article of peduncle dorso-distally slightly produced. Accessory flagellum biarticulated; distal article shorter than one-half of the proximal article. Lengths of antennae I: II as 1.0: 0.50. Flagellum of antenna II with 6–8 articles. Lengths of peduncle articles 4: 5 as 1.0: (0.84–0.95); flagellum 54–70% of peduncle length (articles 4 + 5) (Fig. 5).

Inner lobes of labium longer than half of outer lobes (Fig. 5).

Left mandible: incisor with 5 teeth, lacinia mobilis with 4 teeth; between lacinia and molar 6–9 thick, serrated setae, long seta at base of molar absent (Fig. 5).

Right mandible: incisor processus with 4–5 teeth, lacinia mobilis with several small denticles (more then 12), between lacinia and molar 6–7 thick, serrated setae, long seta at base of molar present. Proportions of mandibular palp articles 2: 3 (distal) as 1.0: 1,20 (1.17–1.32). Proximal palp article without setae; second article with 9–11 seta in 5–6 groups; distal article with 1 group of 3–5 ’A setae’; 3 groups of ’B setae’; 16–24 ’D setae’; 3–5 ’E setae’ (Fig. 5).

Maxilla I distal palp article with 2–3 apical and subapical setae. Outer lobe of maxilla I with 7 spines, 1–3 spines are always pluri-toothed with 3–6 lateral tooth while 4–6 spines are uni-, or bitoothed. Inner lobe with 1–2 setae (Fig. 5).

Maxilla II inner lobe slightly smaller than outer lobe; both of them setose apically and subapically, number of setae is approximately 13–23 per lobe (Fig. 5).

Maxilliped palp article 2 with 11–17 rows of setae along inner margin; distal article with dorsal seta and group of small setae at base of nail. Maxilliped outer lobe with 6–12 flattened, thick setae and 3–8 serrated setae; inner lobe with 2–3 flattened, thick setae apically and 5–9 serrated setae (Fig. 5).

**Figure 6.**
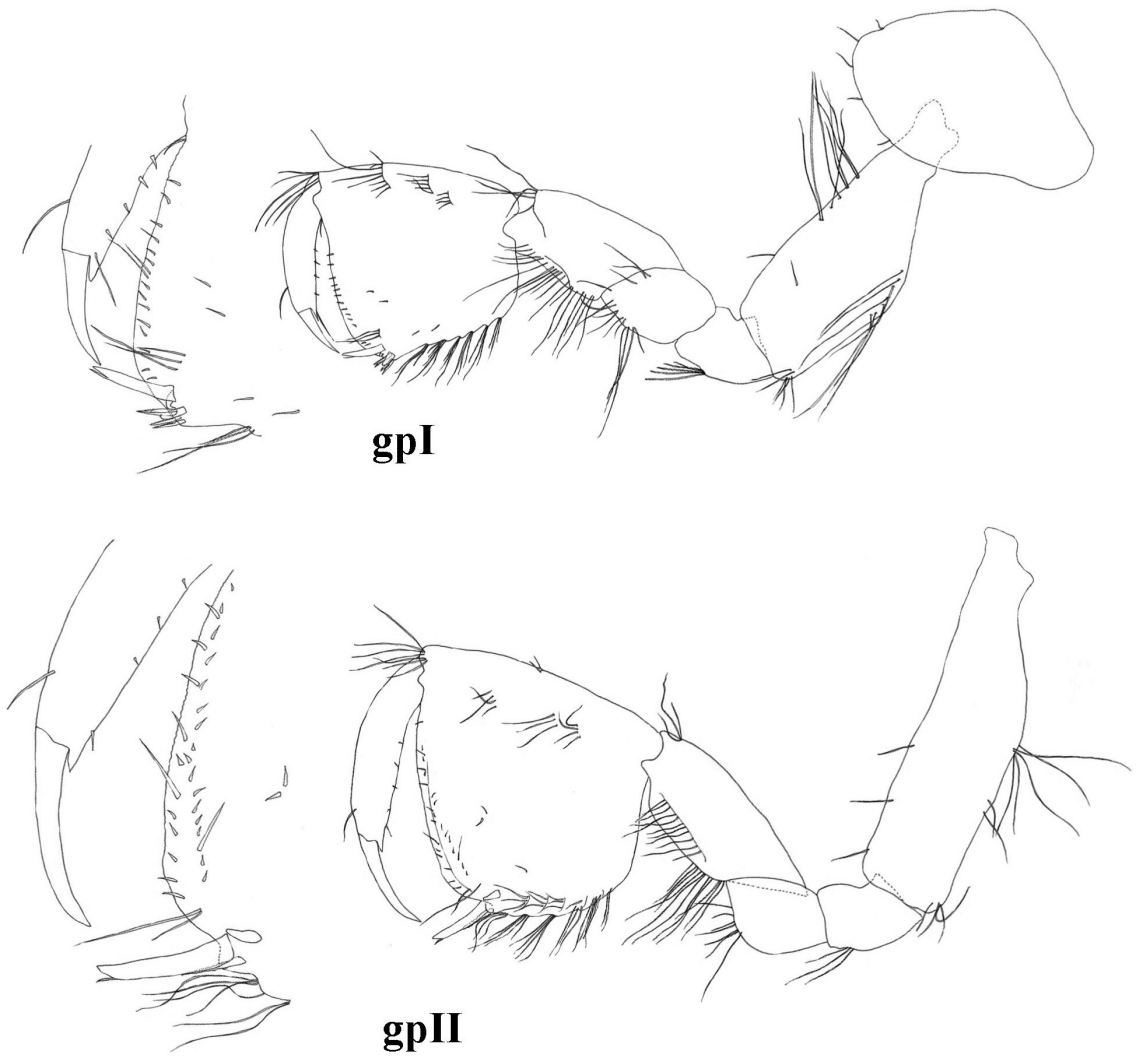
*Niphargus molnari*, gpI = gnathopod I, gpII = gnathopod II.

**Figure 7.**
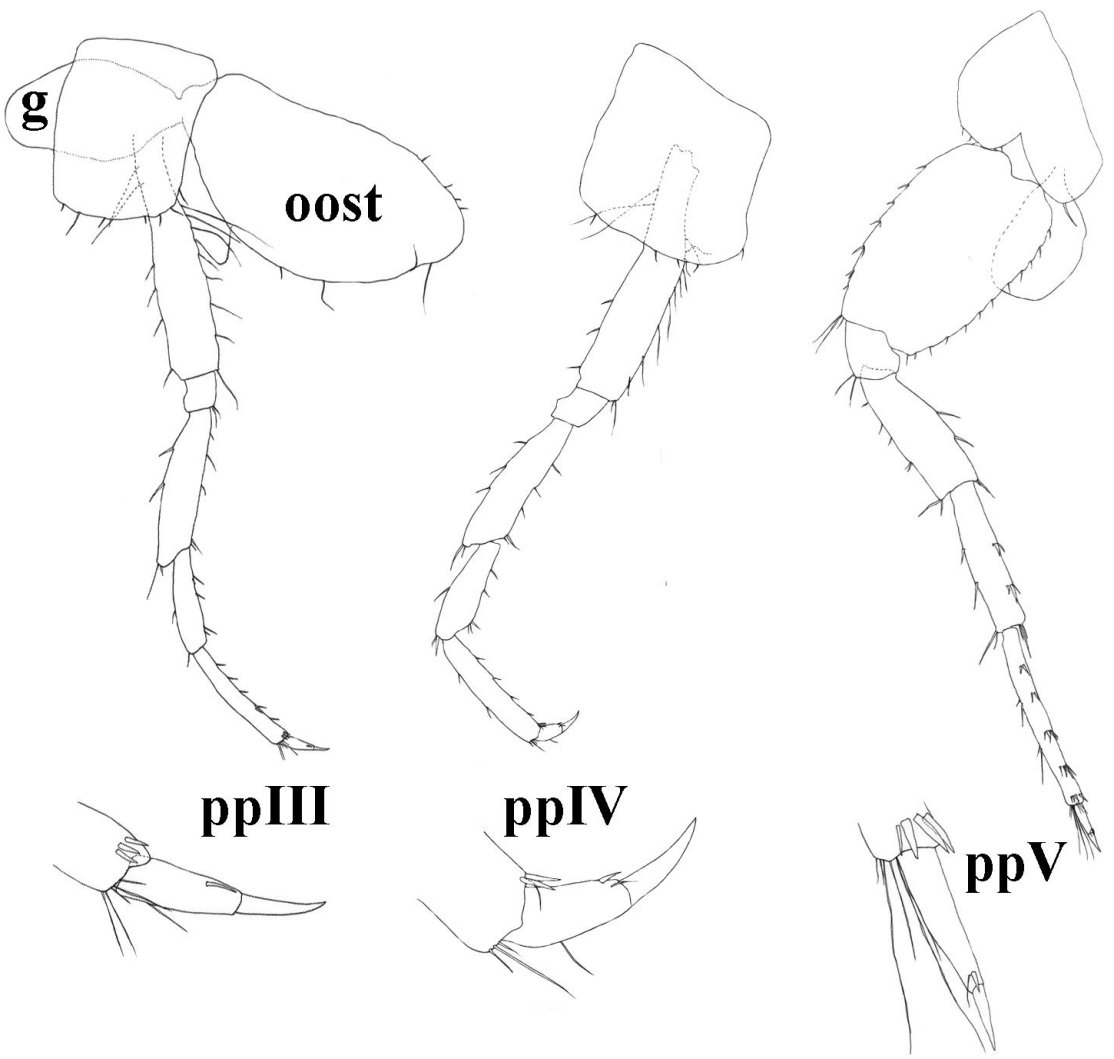
*Niphargus molnari*, ppIII = pereopod III, ppIV = pereopod IV, ppV = pereopod V, g = gill, oost = oostegit.

Coxal plates. Coxal plate I width: depth as 1.00: 1.03 (0.89–1.16), of flattened rhomboid shape, antero-ventral corner subrounded; anterior and ventral margin of coxa I with 3–6 setae (Fig. 6). Coxal plate II width: depth as 1.00: 0.84 (0.76–0.95); anterior and ventral margin with 5–8 setae. Coxal plate III width: depth as 1.00: 0.82 (0.71–1.00); along antero-ventral margin 4–7 setae (Fig. 7). Coxal plate IV width: depth as 1.00: 1.03 (1.26–0.88); posteriorly concave; along antero-ventral margin 5–7 setae (Fig. 7). Coxal plates V-VI: anterior lobe well developed; along posterior margin 1 seta (Fig. 7). Coxal plate VII half-egg shaped, along posterior margin 1 seta (Fig. 7). Gills II-VI ovoid, with approximately same size as coxa VI (Fig. 7).

Gnathopods. Basis width is 38 (33–45)% of basis length. Gnathopod I ischium with 4–8 posterodistal setae in 1 row. Carpus length 62 (57–75)% of basis length and 87 (80–100)% of propodus length. Anterior margin of carpus only with distal group of setae; carpus posteriorly with transverse rows of setae proximally and a row of lateral setae, posterior enlargment small. Propodus subquadrate, palm convex. Along posterior margin 6–8 rows of denticulated setae. Anterior margin with 10–17 setae in 2–3 groups, antero-distal group with 6–12 setae. Group of 2–4 facial setae below (proximal of) palmar spine; 2–4 single surface setae present. Palmar corner with palmar spine, single supporting spine on inner surface, and 3 (rarely 4) denticulated, thick spiniform setae on outer side. Nail length 36 (34–37)% of total dactylus length; along anterior margin single seta; along inner margin 4–5 setae (Fig. 6).

Gnathopod II basis width: length as 1.0: 0.26 (0.21–0.29). Ischium with 2–6 postero-distal setae. Carpus length 56 (50–61)% of basis length and 86 (71–94)% of propodus lenght. Anterior margin of carpus only with distal row of setae; carpus posteriorly with transverse rows of setae proximally, a row of lateral setae; postero-proximal bulge small, positioned proximally. Propodus medium-sized (sum of length, diagonal and palm length measures up to 19 (15–21)% of body length) and larger than propodus of gnathopod I (1.0: 0.57 (0.65–0.85)). Propodus rectangular, palm convex. Posterior margin convex with 6–9 rows of denticulated setae. Anterior margin with 10–20 setae in 3–5 groups; antero-distal group with 7–9 setae. 1 group of 2–3 facial setae below (distal of) palmar spine; 1–4 individual surface setae present. Palmar corner with strong palmar spine, single supporting spine on inner surface, and 1 denticulated, thick spiniform seta on outer side. Nail length 31 (22–36)% of total dactylus length. Along anterior margin single seta; along inner margin 4–6 short setae (Figs 4, 6).

Pereopods III-IV. Proportions of pereopods III: IV as 1: 0.95 (0.93–0.97). Dactylus IV 45 (39–51)% of propodus IV; nail length 47 (39–52)% of total dactylus length. Dactyli III–IV with one dorsal plumose seta, one spine-like seta at the base of the nail, and tiny seta near the spine-like seta (sometimes not visible or absent). Additional spiniform setae on posterior margin are absent (Fig. 7).

**Figure 8.**
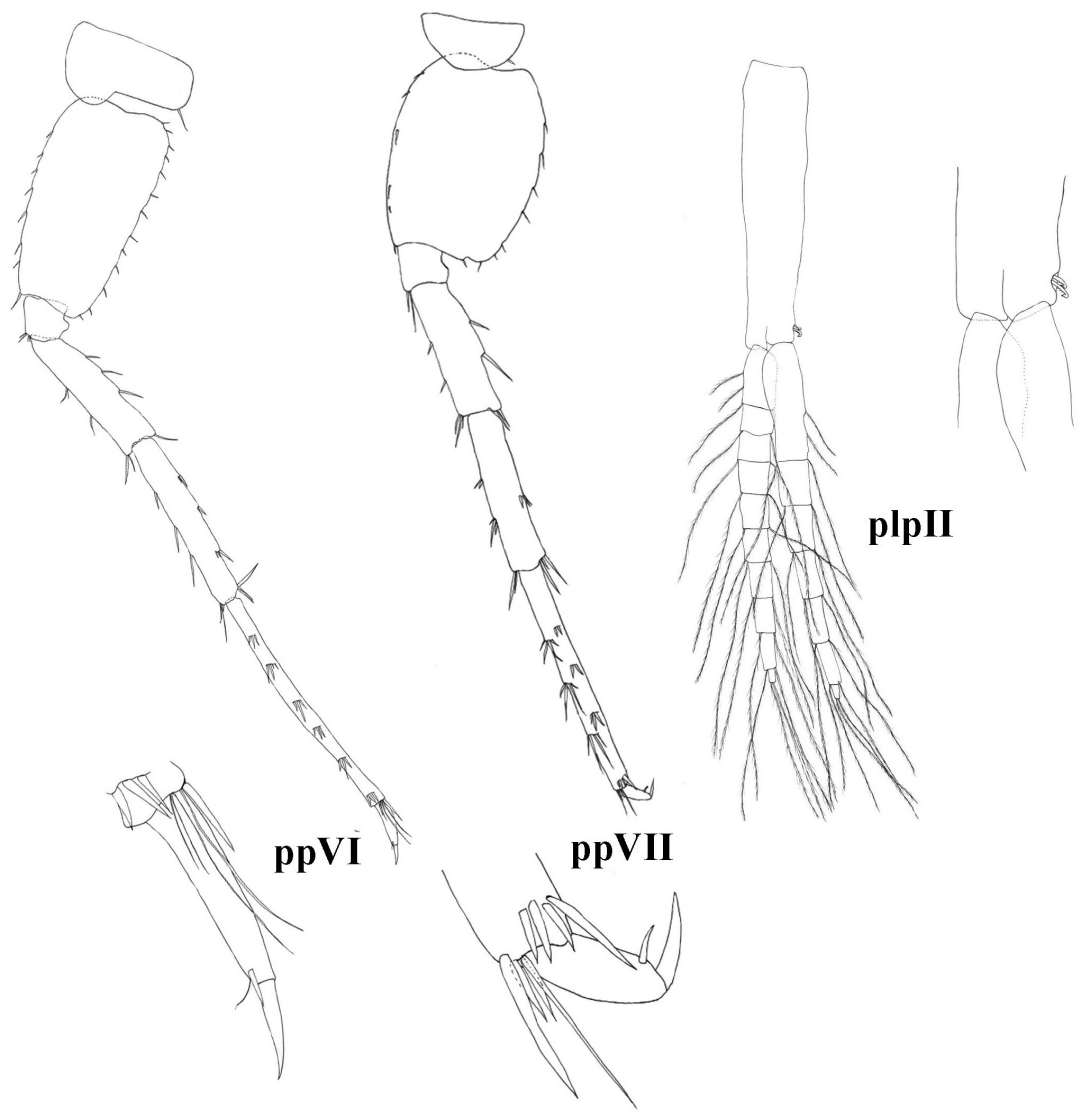
*Niphargus molnari*, ppVI = pereopod VI, ppVII = pereopod VII, plpII = pleopod II.

Pereopods V-VII. Proportions of pereopods V: VI: VII as 1.00: 1.4 (1.37–1.54): 1.5 (1.42–1.61). Pereopod VII length 47 (42–52)% of body length. Basis V-VII narrow with convex posterior margins. Basis V width is 70 (60–78)% of length, basis VI is 67 (59–76)% of length and basis VII is 66 (56–76)% of length. Basis V with small posterodistal lobe, posterior margin with 8-13 setae, anterior margin with 6-8 groups of setae. Dactylus V with one dorsal plumose seta, one spine-like seta at the base of the nail, and tiny seta near the spine-like seta (sometimes not visible or absent). Additional spiniform setae on posterior margin are absent (Fig. 7). Basis VI with small posteriodistal lobe, posterior margin with 9–14 setae, anterior margin with 6–10 setae. Dactylus VI with one dorsal plumose seta (sometimes not visible or absent), one spine-like seta at the base of the nail, and tiny seta near the spine-like seta (sometimes not visible or absent). Additional spiniform setae on posterior margin are absent (Fig. 8).

Basis VII posterior margin with 6–13 setae, anterior margin with 6–11 groups of setae. Total number of basis setae is 15–21. Dactylus VII length 26 (24–29)% of propodus VII length; nail length 26 (16–33)% of total dactylus length. Dactylus VII with one spine-like seta at the base of the nail. Additional spiniform setae on posterior margin are absent (Fig. 8).

Pleopods. Pleopods I-III with 2-hooked retinacles. Pleopod II rami of 16–20 articles each (Figs 4, 8).

**Figure 9.**
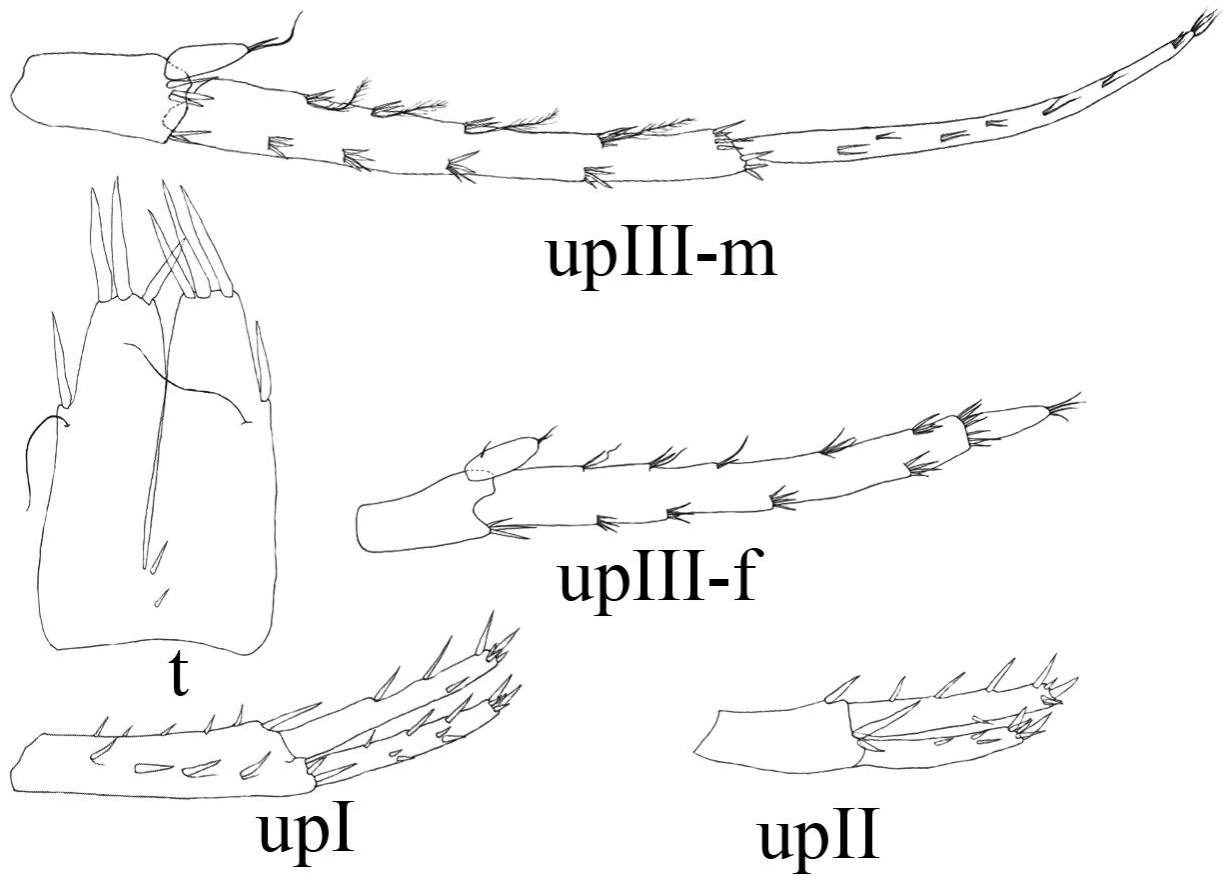
*Niphargus molnari*, t = telson, upI = uropod I, upII = uropod II, upIII-f = female’s uropod III, upIII-m = male’s uropod III.

Uropods. Uropod I basipodite with 6 dorso-lateral and 6 dorsomedial spinifom setae. Length ratio endopodite: exopodite as 1.00: 0.89 (0.83–1.0); rami slightly curved. Endopodite total setae number 2–4 in 2–3 groups, apically 5 spinifom setae. Exopodite with 2–7 spines; apically 5 spinifom setae (Fig. 9).

Uropod II endopodite: exopodite length as 1.00: 0.81 (0.77–0.9) (Fig. 9).

Uropod III up to 38–46% (males) and 12–42% (females) of body length. Basipodite with no lateral seta and 3–6 apical spiniform and thin setae. Endopodite 58–61% (males) and 48–70% (females) of basipodite length, endopodite apically with 1–2 thin-flexible and spiniform setae; laterally 0–1 seta. Exopodite of uropod III rod-shaped, distal article of exopodite 83–115% (males) and 18–73% (females) of proximal exopodite article length. Proximal article with 4–5 groups of plumose, thin-flexible and spiniform setae along inner margin and 4 groups of thin-flexible and spiniform setae along outer margin. Distal article with 3–6 apical setae; lateral setae only in males (Fig. 9).

### Redescription of *Niphargus gebhardti* Schellenberg, 1934 Order Amphipoda Latreille, 1816 Suborder Gammaridea Latreille, 1802 Family Niphargidae G. Karaman, 1962 Genus Niphargus Schiödte, 1849

#### Niphargus gebhardti

Taxon classificationAnimaliaAmphipodaNiphargidae

Schellenberg, 1934

Niphargus foreli gebhardti n. subsp.: Schellenberg 1934; Type locality: Abaligeti Cave. Data from the original description is available in Suppl. material 3.
Niphargus foreli gebhardti : Schellenberg 1935, additional morphological data
Niphargus foreli gebhardti , *Niphargus gebhardti*: Gebhardt 1934, 1963, 1967, distributional data
Niphargus gebhardti : Méhely 1941, morphological data
Niphargus foreli gebhardti : Dudich 1941, distributional data
Niphargus gebhardti : Angyal and Balázs 2013, morphological and distributional data

##### Material examined for redescription.

7 females and 4 males from a permanent pool in the main passage near ’Karthago romjai’ hall of the Abaligeti Cave (Cadastre number: 4120-1, Hungarian Cave Cadastre), collected on 23 March 2013 (leg. D. Angyal & A. Illés), dissected and mounted on slides; additional 4 specimens not dissected. Slides were deposited in the Collection of Crustaceans of the Hungarian Natural History Museum with the following codes: N.GEB-02, N.GEB-03, N.GEB-04, N.GEB-05, N.GEB-08, N.GEB-10, N.GEB-14, N.GEB-15, N.GEB-17, N:GEB-18, N.GEB-20. Diagnostic voucher numbers of specimens used for molecular studies: NB 550 (*Niphargus gebgardti*, coll. data: Abaligeti Cave, main passage, pool, 23 March 2013, leg. D. Angyal & A. Illés), NB 551 (*Niphargus gebgardti*, coll. data: Szajha-felső Sinkhole (Cadastre number: 4120-16), small pool, 2 April 2013, leg. D. Angyal & Z. Tegzes).

COI Gen Bank Accession Numbers: KP967553 (Abaligeti Cave), KP967554 (Szajha-felső Sinkhole)

##### Diagnosis.

Small-sized niphargid; epimeral plate III postero-ventral corner subrounded. Telson with 3–6 apical spines, 0–2 lateral spines, 0–1 lateral plumose setae, 0–1 spines in cleft and 0–1 dorsal surface spines. Maxilla I outer lobe with 7 spines, pluri-, uni-, bi-toothed spines alternating. Gnathopod I and gnathopod II dactyli with single seta on outer margin. Gills II-VI ovoid. Pleopods I-III with 3, rarely 4 retinacles on each. Uropod I lenght of endopodite: length of exopodite ratio as 1.00: (1.09–1.11) on males and 1.00: (1.03–1.17) on females. Uropod II sexually dimorphic, exopodite rod-shaped, distal article of exopodite on males 95–155% of proximal article length and 52–72% on females.

##### Description.

**Figure 10.**
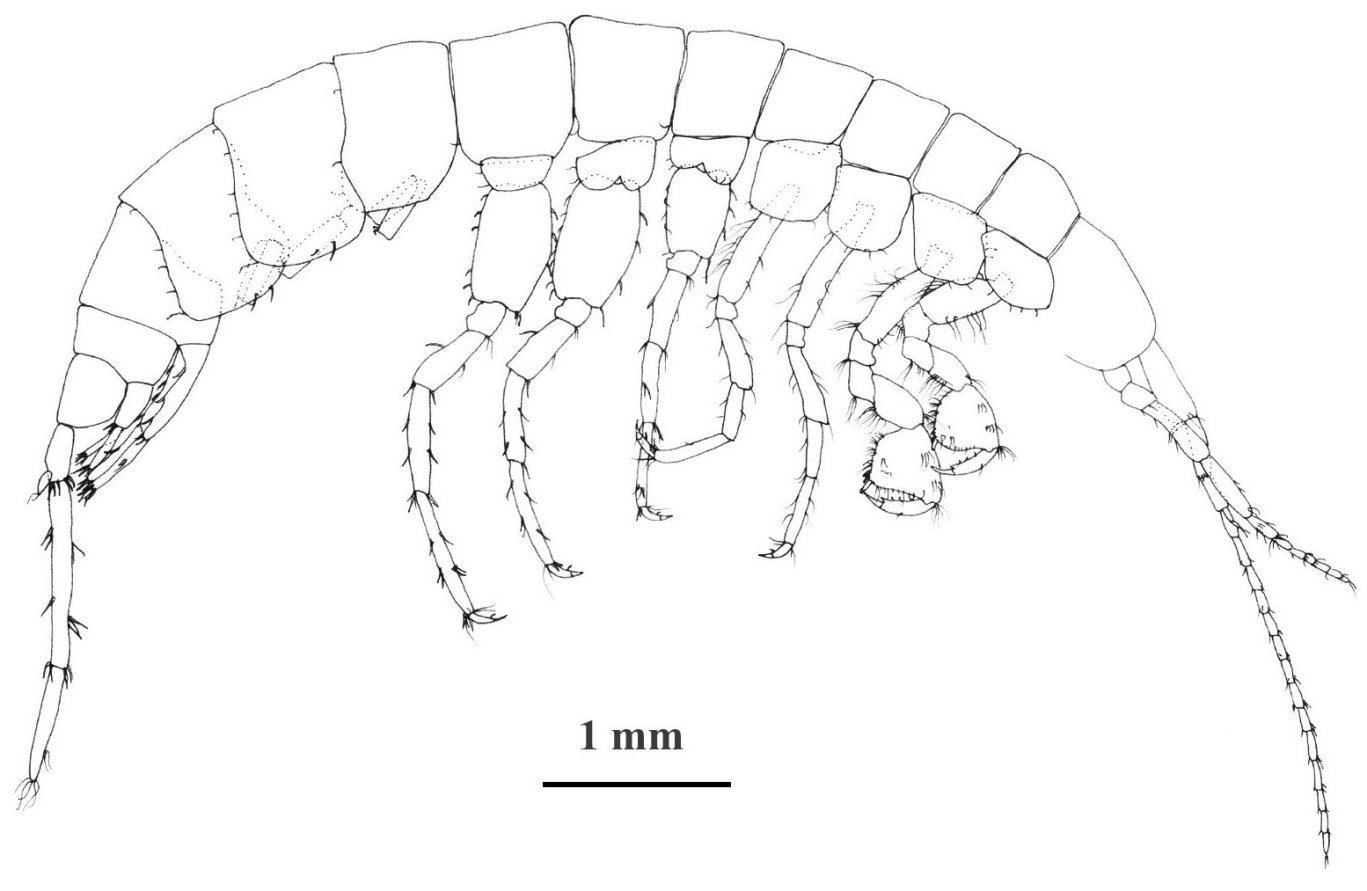
*Niphargus gebhardti*, female from the Abaligeti Cave, lateral view. Mouthparts, rami of pleopods and telson are not drawn.

**Figure 11.**
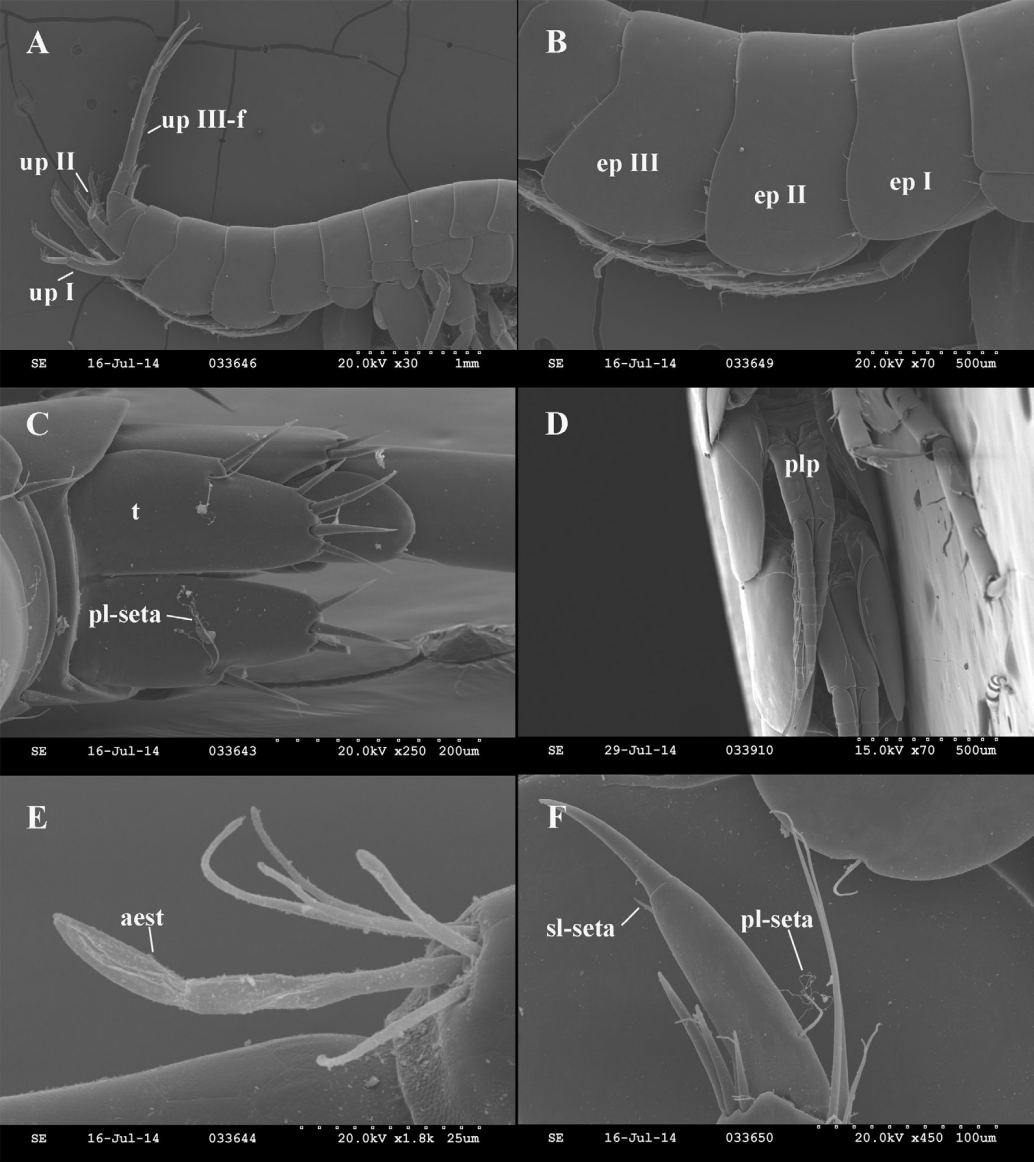
*Niphargus gebhardti*, scanning electron micrographs. **A** epimeral plates with uropods (Ep1–3 = epimeral plates 1–3, upI = uropod I, upII = uropod II, upIII-f = female’s uropod III) **B** epimeral plates (Ep1–3 = epimeral plates 1–3) **C** telson (t = telson, pl-seta = plumose seta) **D** pleopods (plp = pleopod) **E** aesthetasc on antenna I (aest = aesthetasc) **F** pereopod VI dactylus (sl-seta = spine-like seta at the base of the nail, pl-seta = plumose seta).

Body and telson. Small-sized niphargid species, females 4.9–5.9 mm, males 5.9–7.0 mm. Head length up to 9% of body length; rostrum absent. Pereonites I-VI without setae; pereonite V, VI, VII with 1 postero-ventral seta each. Pleonites I-III with 1–2 setae along dorso-posterior margin. Epimeral plate II posterior and ventral margins convex, ventro-postero-distal corner rounded. Along ventral margin 1–3 spiniform setae; along posterior margin 3–4 thin setae. Epimeral plate III ventral and posterior margins convex, ventro-postero-distal corner rounded; along ventral margin 2–3 spiniform setae; along posterior margin 4 thin setae. Urosomite I postero-dorso-laterally with 1 seta; urosomite II postero-dorso-laterally with 1 spiniform seta; urosomite III without setae. Near insertion of uropod I 1 spiniform seta (Figs 10, 11).

Telson length: width as 1.0: 0.88; cleft 74 (70–79)% of length; lobes apically widely rounded. Telson spines (per lobe): 2–4 apical spines, 33.5 (28–39)% of telson length; lateral margins with 0–2 spine and 0–1 plumose setae; 0–1 in cleft spines, 0 or 1 dorsal surface spines, 1 basal spine (Figs 11, 16).

**Figure 12.**
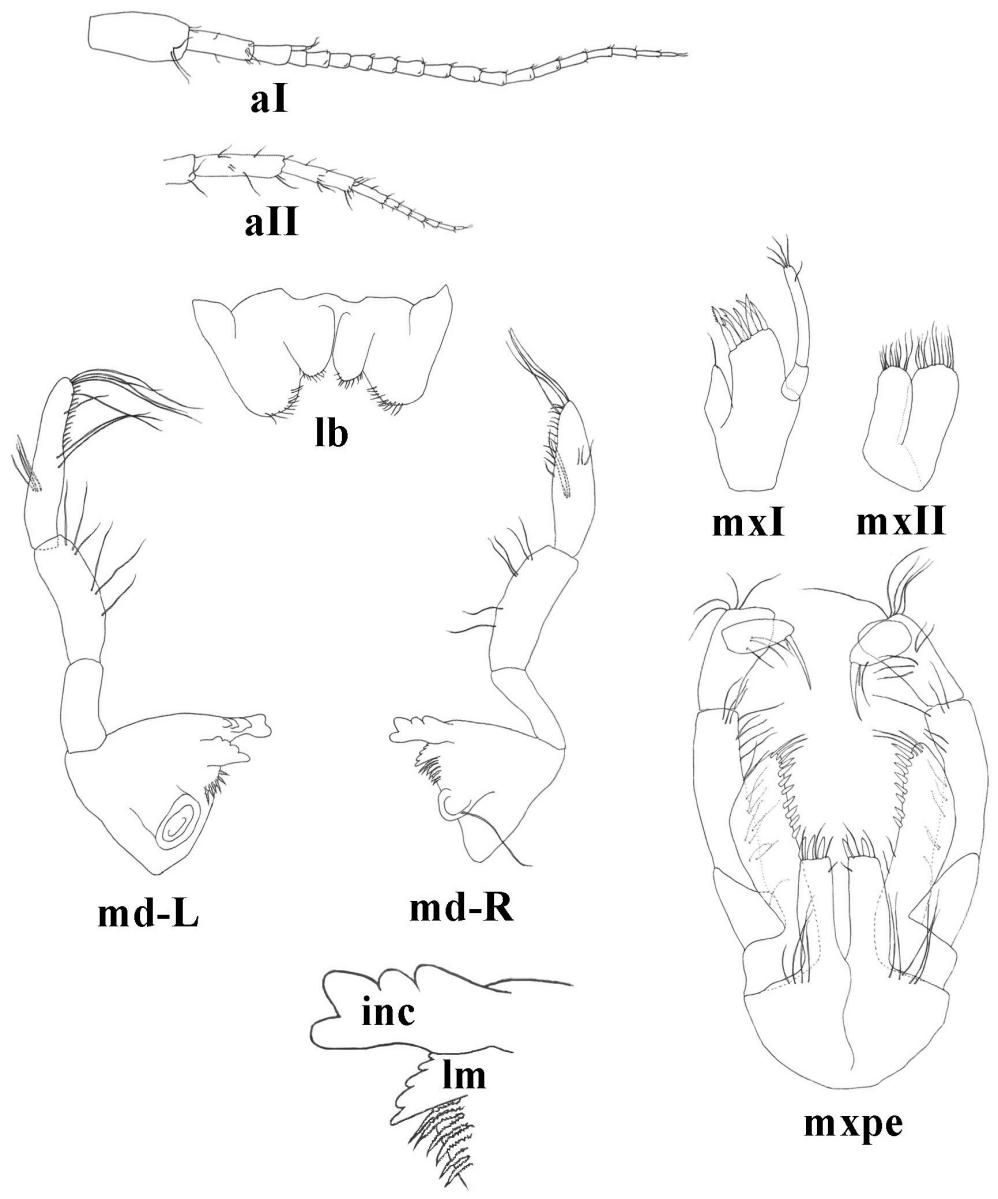
*Niphargus gebhardti*, aI = antenna I, aII = antenna II, mxI = maxilla I, mxII = maxilla II, md-R = right mandibula, inc = incisor, lm = lacinia mobilis, md-L = left mandibula, lb = labium, mxpe = maxilliped.

Antennae and mouthparts. Antenna I 37 (34–41)% of body length. Flagellum with up to 13–16 articles; each article with 1 long aesthetasc (Fig. 11). Peduncle article 1: 2: 3 as 1.0: 0.69 (0.60–0.76): 0.37 (0.30–0.4). Proximal article of peduncle dorso-distally slightly produced. Accessory flagellum biarticulated; distal article 52 (38–67)% of proximal article. Lengths of antennae I: II as 1.0: 0.48 (0.42–0.52). Flagellum of antenna II with 6–8 articles. Lengths of peduncle articles 4: 5 as 1.0: 0.85 (0.81–0.91); flagellum 73 (57–81)% of peduncle length (articles 4+5) (Fig. 12).

Inner lobes of labium longer than half of outer lobes (Fig. 12).

Left mandible: incisor with 5 teeth, lacinia mobilis with 4 teeth; between lacinia and molar 5–7 thick, serrated setae, long seta at base of molar absent (Fig. 12).

Right mandible: incisor processus with 4 teeth, lacinia mobilis with 5–6 denticles, between lacinia and molar 6–8 thick, serrated setae, 1 long seta at base of molar present. Proportions of mandibular palp articles 2: 3 (middle: distal) as 1.0: 1.1 (1.00–1.21). Proximal palp article without setae; second article with 4–6 seta in 3–4 groups; distal article with 1 group of 3–4 ’A setae’; 2–4 of ’B setae’ (single or in groups); 9–13 ’D setae’ and 3–5 ’E setae’ (Fig. 12).

Maxilla I distal palp article with 3–6 apical and subapical setae. Outer lobe of maxilla I with 7 spines, pluri-, uni-, bi-toothed spines alternating. Inner lobe with 1 seta (Fig. 12).

Maxilla II inner lobe slightly smaller than outer lobe; both of them setose apically and subapically, number of setae is approximately 6–11 on inner lobe and 8–12 on outer lobe (Fig. 12).

Maxilliped palp article 2 with 8–11 rows of setae along inner margin; distal article with dorsal seta and group of small setae at base of nail. Maxilliped outer lobe with 6–8 flattened, thick setae and 3–5 serrated setae; inner lobe with 2–3 flattened, thick setae apically and 2–4 serrated setae (Fig. 12).

**Figure 13.**
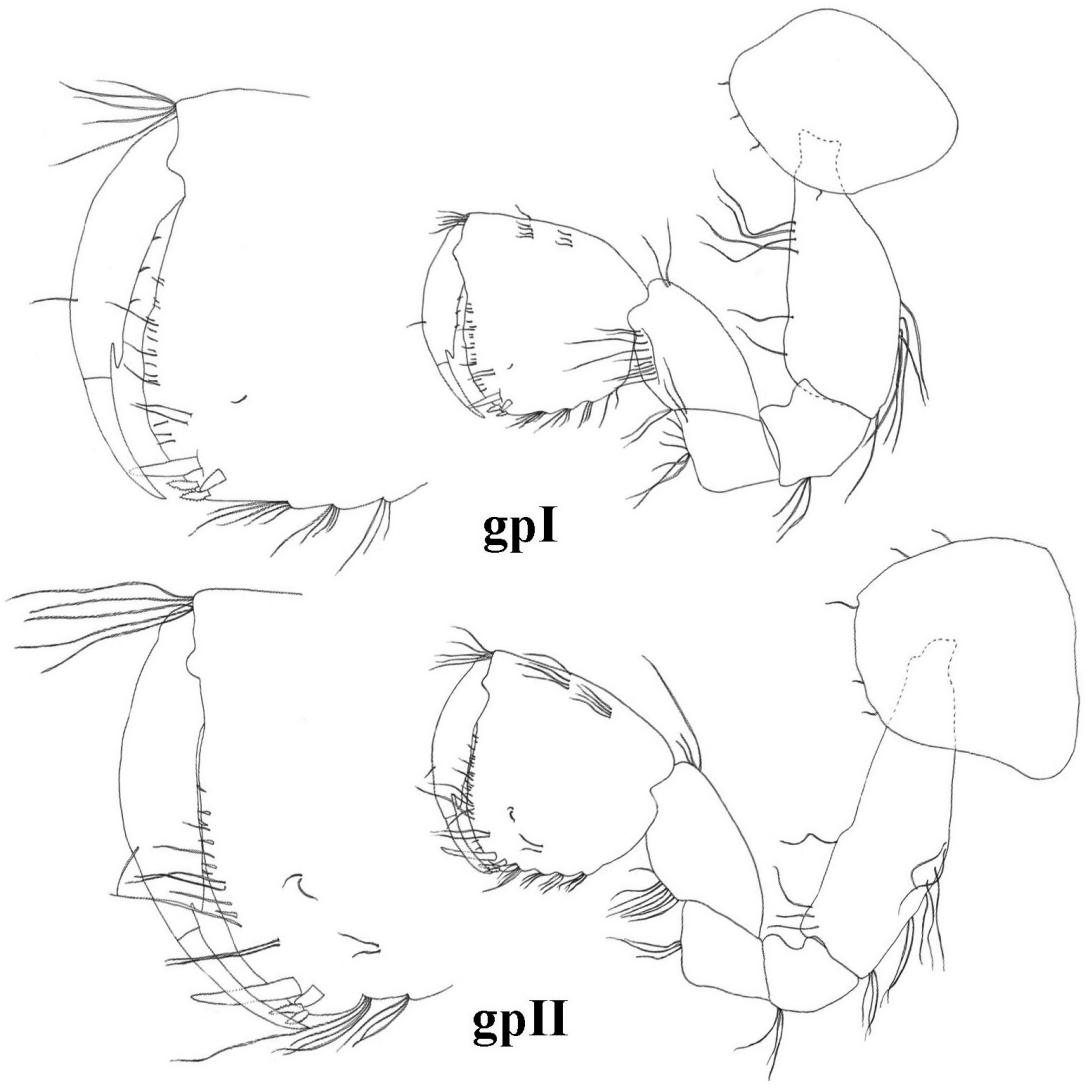
*Niphargus gebhardti*, gpI = gnathopod I, gpII = gnathopod II.

**Figure 14.**
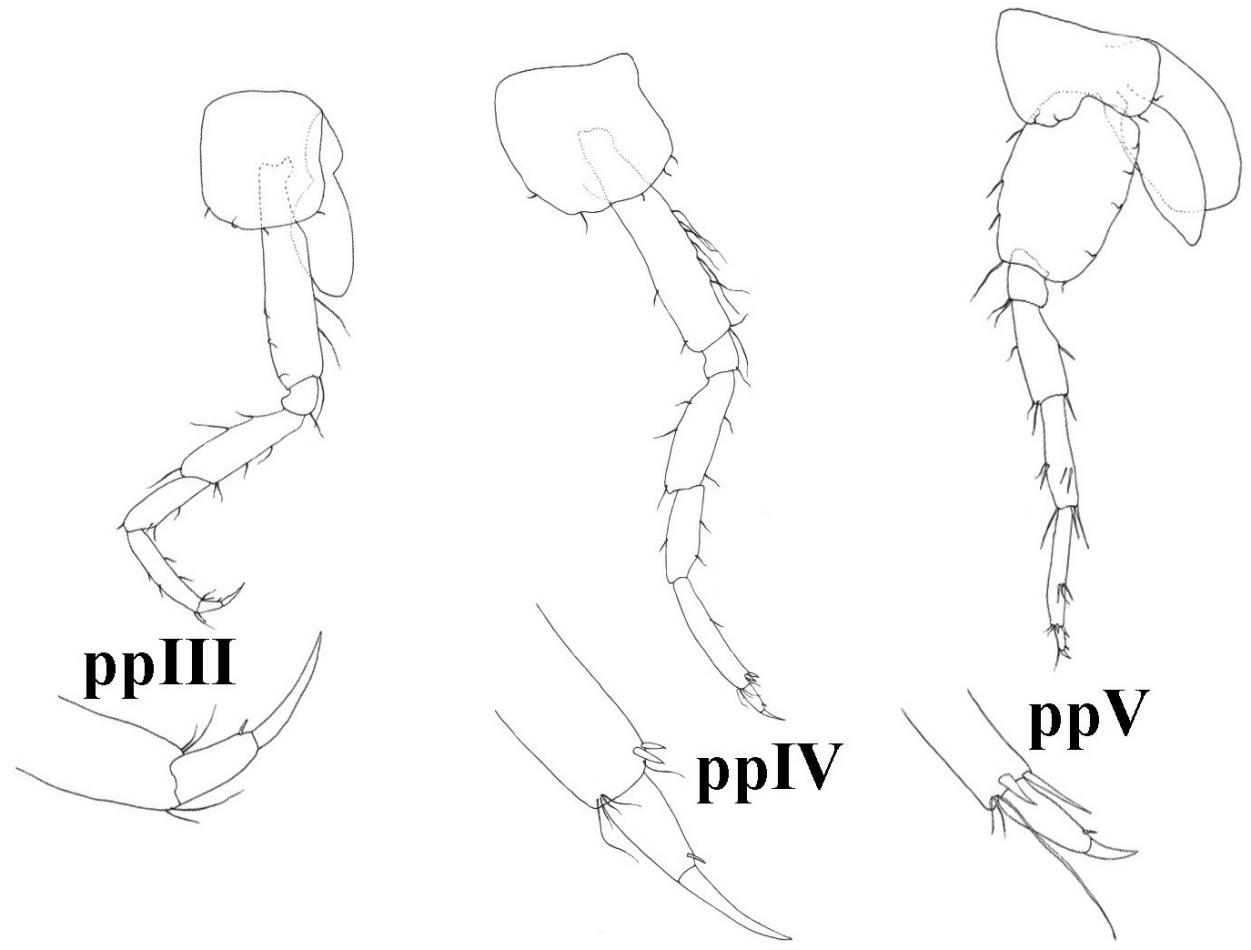
*Niphargus gebhardti*, ppIII = pereopod III, ppIV = pereopod IV, ppV = pereopod V.

**Figure 15.**
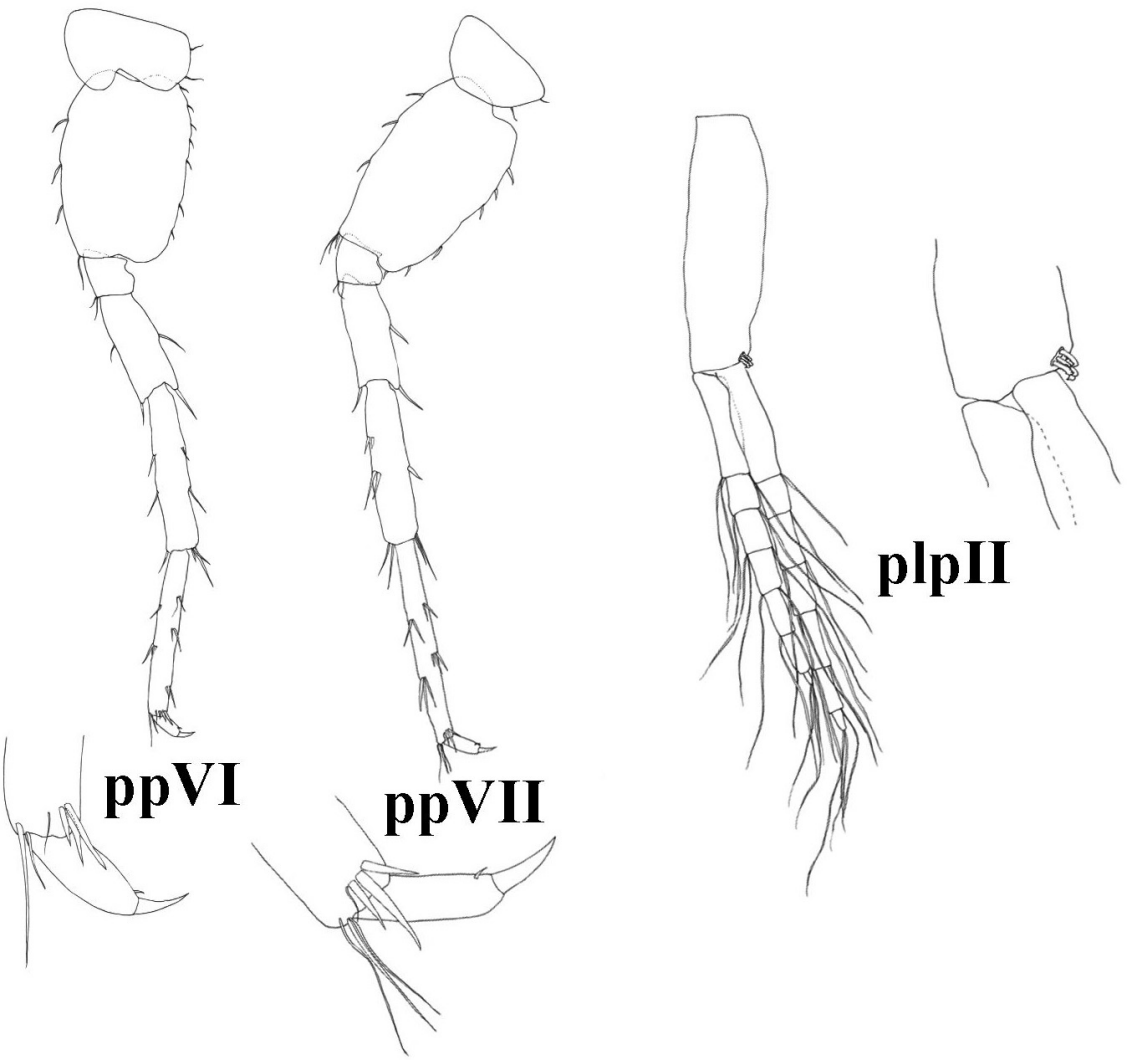
*Niphargus gebhardti*, ppVI = pereopod VI, ppVII = pereopod VII, plpII = pleopod II.

Coxal plates. Coxal plate I width: depth as 1.00: 0.76 (0.6–0.9) of flattened rhomboid shape, antero-ventral corner subrounded; anterior and ventral margin of coxa I with 4–6 setae (Fig. 13). Coxal plate II width: depth as 1.00: 0.97 (0.83–1.21); anterior and ventral margin with 3–6 setae (Fig. 13). Coxal plate III width: depth as 1.00: 1.12 (1.05–1.2); along antero-ventral margin 4–6 setae. Coxal plate IV width: depth as 1.00: 1.04 (0.97–1.12); posteriorly concave; along antero-ventral margin 4–5 setae (Fig. 14). Coxal plates V-VI with well developed anterior lobe, and smaller posterior lobe with usually 2 setae (occasionally with 1 or 3) in postero-ventral corner. Coxal plate VII half-egg shaped, along posterior margin 2 setae. Gills II-VI ovoid, of approximately similar size as coxa VI (Fig. 15).

Gnathopods. Gnathopod I basis width 42 (38–47)% of basis length. Ischium with 3–4 posterodistal setae in 1 row. Carpus length 61 (52–82)% of basis length and 98 (87–110)% of propodus length. Anterior margin of carpus only with distal group of setae; carpus posteriorly with transverse rows of setae proximally and a row of lateral setae, posterior enlargment small. Propodus subquadrate, palm and posterior margin convex. Along posterior margin 3–4 rows of denticulated setae. Anterior margin with 6–11 setae in 2–3 groups, antero-distal group with 4–8 setae. Group of 2–3 facial setae below (proximal of) palmar spine; 1–4 surface setae in 1–2 groups present. Palmar corner with palmar spine, single supporting spine on inner surface, and 2–3 denticulated, thick spiniform setae on outer side. Nail length 33 (30–39)% of total dactylus length; along anterior margin single seta; along inner margin 3–4 setae (Fig. 13).

Gnathopod II basis width: length as 1.0: 0.34 (0.27–0.45). Ischium with 3–4 postero-distal setae in 1 row. Carpus length 59 (48–69)% of basis length and 106 (96–111)% of propodus length. Anterior margin of carpus only with distal row of setae; carpus posteriorly with transverse rows of setae, proximally a row of lateral setae; postero-proximal bulge small and positioned proximally. Propodus small to medium-sized (sum of length, diagonal and palm length measures up to 12–15% of body length) and larger than propodus of gnathopod I (1.0: 0.87 (0.78–0.96)). Propodus rectangular, palm convex. Posterior margin straight or convex with 4–5 rows of denticulated setae. Anterior margin with 3–9 setae in 1–2 groups; antero-distal group with 4–8 setae. Group of 2–4 facial setae below (proximal of) palmar spine; 2–3 surface setae in 1–2 groups present. Palmar corner with strong palmar spine, single supporting spine on inner surface, and 2–3 denticulated, thick spiniform setae on outer side. Nail length 34 (29–42)% of total dactylus length. Along anterior margin single seta; along inner margin 3 short setae (Fig. 13).

Pereopods III–IV. Proportions of pereopods III: IV as 1: 0.96 (0.89–1). Dactylus IV 51 (46–57)% of propodus IV lenght; nail length 53 (44–61)% of total dactylus length. Dactyli III-IV with dorsal plumose seta (sometimes not visible or absent), one spine-like seta at the base of the nail, and tiny seta near the spine-like seta (sometimes not visible or absent). Additional spiniform setae on posterior margin are absent (Fig. 14).

Pereopods V–VII. Proportions of pereopods V: VI: VII as 1.00: 1.3 (1.27–1.49): 1.5 (1.46–1.58). Pereopod VII length 42–45% of body length. Basis V-VII with convex posterior margins. Basis V width is 71 (66–80)% of length, basis VI is 68 (64–73)% of length, and basis VII is 66 (63–69)% of length. Basis V with small posterodistal lobe, posterior margin with 4–6 setae, anterior margin with 4–9 setae in 3+1 groups (Fig. 14). Pereopod dactylus V with one dorsal plumose seta (sometimes not visible or absent), and one spine-like seta at the base of the nail (Fig. 14). Basis VI with small posterodistal lobe, posterior margin with 6-7 setae, anterior margin with 5-8 setae in 3-4 groups. Dactylus VI with one spine-like seta at the base of the nail, and tiny seta near the spine-like seta (sometimes not visible or absent). Additional spiniform setae on posterior margin are absent (Fig. 15). Basis VII posterior margin with 5–8 setae, anterior margin with 3–5 groups of setae. Total number of basis setae is 11–15. Dactylus VII length 26 (23–35)% of propodus VII length; nail length 28.5 (25–38)% of total dactylus length. Dactyli VI with one dorsal plumose seta (sometimes not visible or absent), one spine-like seta at the base of the nail, and tiny seta near the spine-like seta (sometimes not visible or absent). Additional spiniform setae on posterior margin are absent (Fig. 15).

Pleopods. Pleopods I-III with 3, rarely 4 hooked retinacles. Pleopod II rami of 11–13 articles each (Figs 11, 15).

**Figure 16.**
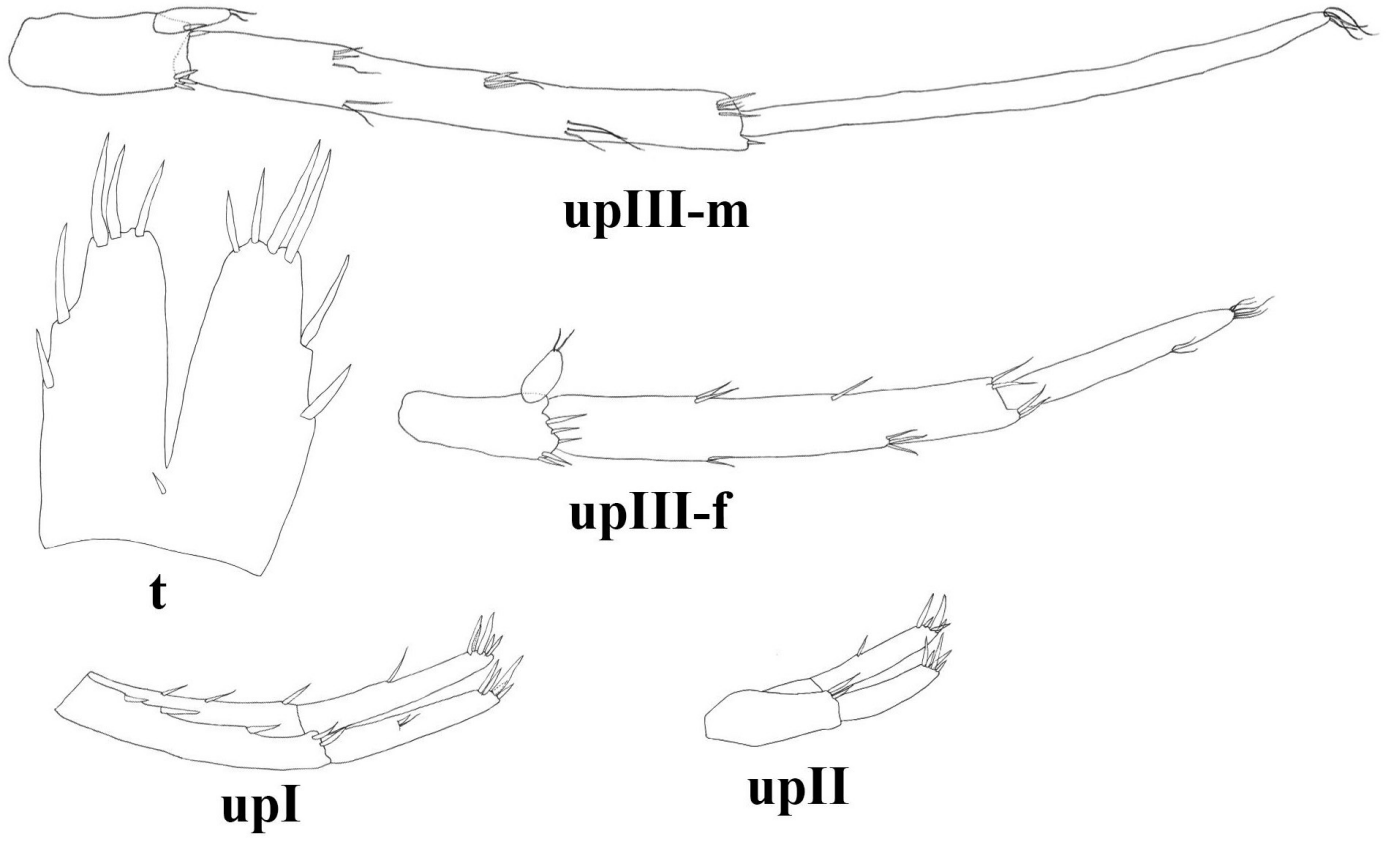
*Niphargus gebhardti*, t: telson, upI: uropod I, upII: uropod II, upIII-m: male’s uropod III, upIII-f: female’s uropod III.

Uropods. Uropod I basipododite with 4–5 dorso-lateral and 1–3 dorsomedial spiniform setae including spiniform setae in distal position. Length ratio endopodite: exopodite as 1.00: 0.91 (0.87–0.97); rami slightly curved. Endopodite with 1–2 setae, apically 5 spinifom setae. Exopodite with 1–4 setae or spines in 1–2 groups; apically 5 spinifom setae (Figs 11, 16).

Uropod II endopodite: exopodite length as 1.00: 0.84 (0.77–0.95) (Figs 11, 16).

Uropod III 38 (37–39)% (males) and 26 (24–30)% (females) of body length. Basipodite with 0–1 lateral setae and 5–6 apical spiniform and thin setae. Endopodite 41 (39–44)% (males) and 48 (41–54)% (females) of basipodite length; endopodite apically with 0–2 thin-flexible and spiniform setae; laterally with 0–1 seta. Exopodite of uropod III rod-shaped, distal article of exopodite 100 (95–105)% (males) or 60 (52–78)% (females) of proximal article length. Proximal article with 3–4 groups of plumose, thin-flexible and spiniform setae along inner margin and 2–4 groups of thin-flexible and spiniform setae along outer margin. Distal article without lateral seta (males) or with 3 setae in 1 group (females); apically 4–7 setae (Figs 11, 16).

### Comparison with phylogenetically related and geographically close species

*Niphargus molnari* and *Niphargus gebhardti* share few main traits (the same body size class, slender body, sexually dimorphic uropod III but not uropod I), but differ from each other in the shape of epimeral plates, the size of gnathopod propodi, in denticulation of spines on outer lobe of maxilla I and in the number of retinacles (Angyal and Balázs 2013). Keeping these differences in mind we compare both species to the species that are either closely related according to molecular phylogeny, or to the species that live in the same geographic area.

*Niphargus vadimi* Birstein, 1961 is known from Crimea. Despite its close position suggested by the presented molecular tree, this species differs from phylogenetically related *Niphargus gebhardti* and non-related *Niphargus molnari* in considerably larger body size and much larger gnathopods.

High morphological similarity to the focal pair of species reveal another four species phylogenetically related to *Niphargus gebhardti*, namely *Niphargus bihorensis* Schellenberg, 1940, *Niphargus fongi* Fišer & Zagmajster, 2009, *Niphargus carniolicus* Sket, 1960, and *Niphargus dobati* Sket, 1999. Epikarstic *Niphargus bihorensis* is known from Romania and Italy, whereas the latter three are known from epikarst and karst river beds from Slovenian caves. All four species share with focal species main traits (body size, slender body, sexually dimorphic uropod III but not uropod I).

*Niphargus bihorensis* and *Niphargus fongi* differ from the focal species in the shape of gills (being narrow instead of ovoid as in focal species) and in higher number of retinacles on pleopods. In addition, *Niphargus fongi* differs from *Niphargus molnari* and *Niphargus gebhardti* by (i) the elevated number of setae along posterior margin of epimeral plate III, (ii) the longer apical telson spines, (iii) and the reduced number of denticulated spines in palmar corners of both gnathopods. *Niphargus bihorensis*, which is a complex of at least two morphologically indistinguishable species (Meleg et al. 2013), differs from the focal species by (i) reduced number of spines on maxilla I outer lobe (only 6), (ii) more numerous setae on maxilla I palpus (7–8), (iii) and by more numerous retinacles.

*Niphargus carniolicus* and *Niphargus dobati* differ from the focal pair of species in the length of rami of uropod I (expopodite equal to or slightly longer than endopodite *versus* exopodite consistently shorter to endopodite in focal species). In addition, *Niphargus carniolicus* differs from *Niphargus molnari* and *Niphargus gebhardti* by (i) shorter apical spines on telson, and (ii) fewer denticulated spines on palmar corner of gnathopods. *Niphargus dobati* differs from the two focal species by (i) the elevated number of spines on uropod I basipodite, (ii) the length of pereopod V and VI (which are longer comparing with pereopod VII), and the (iii) elevated number of mandibular palp ‘D seta’.

Phylogenetic relationship of *Niphargus molnari* to the rest of *Niphargus* species is not clear, however a few morphologically similar species, like *Niphargus schellenbergi* S. Karaman, 1932 are known. It differs from *Niphargus molnari* and *Niphargus gebhardti* by (i) the differently ornamented telson (5–7 long apical spines and 2–5 lateral spines in *Niphargus schellenbergi*, respectively), (ii) more numerous apical setae on uropod III endopodit, (iii) elevated number of pleopod retinaculi, (iv) by the length of uropod I exopodite, which is slightly longer than endopodit, (v) by several setae along outer margin of gnathopod dactyli, and (vi) by bigger body size (>10 mm).

The following species are compared with *Niphargus molnari* and *Niphargus gebhardti* due to their geographical vicinity. *Niphargus forroi* G. Karaman, 1986 was described from Northeast Hungary, and is known from only a couple of caves from the Bükk Mts. Beside the close body size, *Niphargus forroi* agree with *Niphargus molnari* by the similar seta numbers and arrangement on the gnathopods, by the telson spine-pattern, as well as by the number of different spine and seta types on pereopod dactyli. *Niphargus forroi* differs from *Niphargus molnari* by (i) the subrounded posteroventral corner of the epimeral plates, (ii) the lower number of mandibular palp ‘D setae’ and by (iii) the reduced number of maxilla distal article apical seta. *Niphargus forroi* differs from both *Niphargus molnari* and *Niphargus gebhardti* by the number of posterior margin setae on pereopods V-VII. The description of *Niphargus hungaricus* Méhely, 1937 (endemic species of the Kőszegi Mts.) contains no drawings and not enough characters that would be needed for proper comparison. A later work of Méhely (1941) is only partially filling this gap by containing a drawing on the first gnathopod and some additional data on its seta arrangement. According to the available information, *Niphargus hungaricus* differs from *Niphargus molnari* and *Niphargus gebhardti* by (i) the setae number of gnathopods dactyli outer margin (always more than 1 seta of *Niphargus hungaricus*) and by (ii) the length of male’s uropod I endopodite (inner ramus is elongated and two times long as outer ramus in *Niphargus hungaricus*). There are different *Niphargus* populations in the Bükk Mts. and in the Aggtelek Karst belonging to the *Niphargus tatrensis* Wrzesniowsky, 1888 species group including *Niphargus aggtelekiensis* Dudich, 1932. Although the taxonomic status of these populations is not clear, the complex shares several distinct morphological characters that can be compared with the focal species. Populations of *Niphargus tatrensis* – *Niphargus aggtelekiensis* complex differ from *Niphargus molnari* and *Niphargus gebhardti* by (i) larger body size (>15 mm), (ii) the elevated number of setae along outer margin of gnathopods dactyli (there are more than one), (iii) the lower mandibular ‘A’ and ‘D seta’ number and (iv) the elongated distal article of uropod III of both gender. Main diagnostic characters are presented in Table 1.

**Table 1.** Comparison of the main diagnostic characters of *Niphargus molnari*, *Niphargus gebhardti* and the phylogenetically related and geographically close species.

Species	No. apical telson spines	No. lateral telson spines	Pleopod I. no. hooks in retinacle	Pleopod II. no. hooks in retinacle	Pleopod III. no. hooks in retinacle	Uropod I endopodite/exopodite length	Gnathopod dactylus anterior margin seta no.	Shape of gills II-IV	Epimeral plates postero-ventral corner shape	Source of data
*Niphargus molnari* Méhely, 1927	3–4	1–3	2	2	2	endopodite slightly longer	single	ovoid	sharply inclined	own slides
*Niphargus gebhardti* Schellenberg, 1934	3–6	0–2	3 (rarely 4)	3 (rarely 4)	3 (rarely 4)	endopodite slightly longer	single	ovoid	subrounded	own slides
*Niphargus carniolicus* Sket, 1960	4–5	1–2	4–5	4–5	4–5	exopodite slightly longer	single	?	subrounded	Sket 1960, G. Karaman 1989
*Niphargus dobati* Sket, 1999	3+1	2	3–4	3–4	3–4	nearly equal	single	narrow	subrounded	Sket 1999
*Niphargus vadimi* Birstein, 1960	?	3	?	?	?	?	?	?	sharply inclined	Birstein 1961
*Niphargus fongi* Fišer & Zagmajster, 2009	3–5	1–2	4–7	3–5	4–5	equal	single	narrow	subrounded	Fišer and Zagmajster 2009
*Niphargus bihorensis* Schellenberg, 1940	5–7	1 pair, plumose	4–6	4–6	4–6	exopodite slightly longer	single	long and recurved	I., II. subrounded, III. angular	G. Karaman 1980
*Niphargus schellenbergi* S. Karaman, 1932	5–7	2–5	4–6	3–5	3–6	exopodite slightly longer	more than 1	?	subrounded	S. Karaman 1932
*Niphargus forroi* G. Karaman, 1986	2	2	2	2	2	endopodite longer	single	narrow	subrounded	G. Karaman and Ruffo 1986
*Niphargus hungaricus* Méhely, 1937	3–5	1–2	?	?	?	endopodite 2x longer	more than 1	?	subrounded	Méhely 1937, 1941
*Niphargus tatrensis* Wrzesniowsky, 1888	3–4	0–3	2	2	2	nearly equal	more than 1	large, irregularly ovoid	III. sharply inclined	Fišer et al. 2010

### Molecular taxonomy

**Figure 17.**
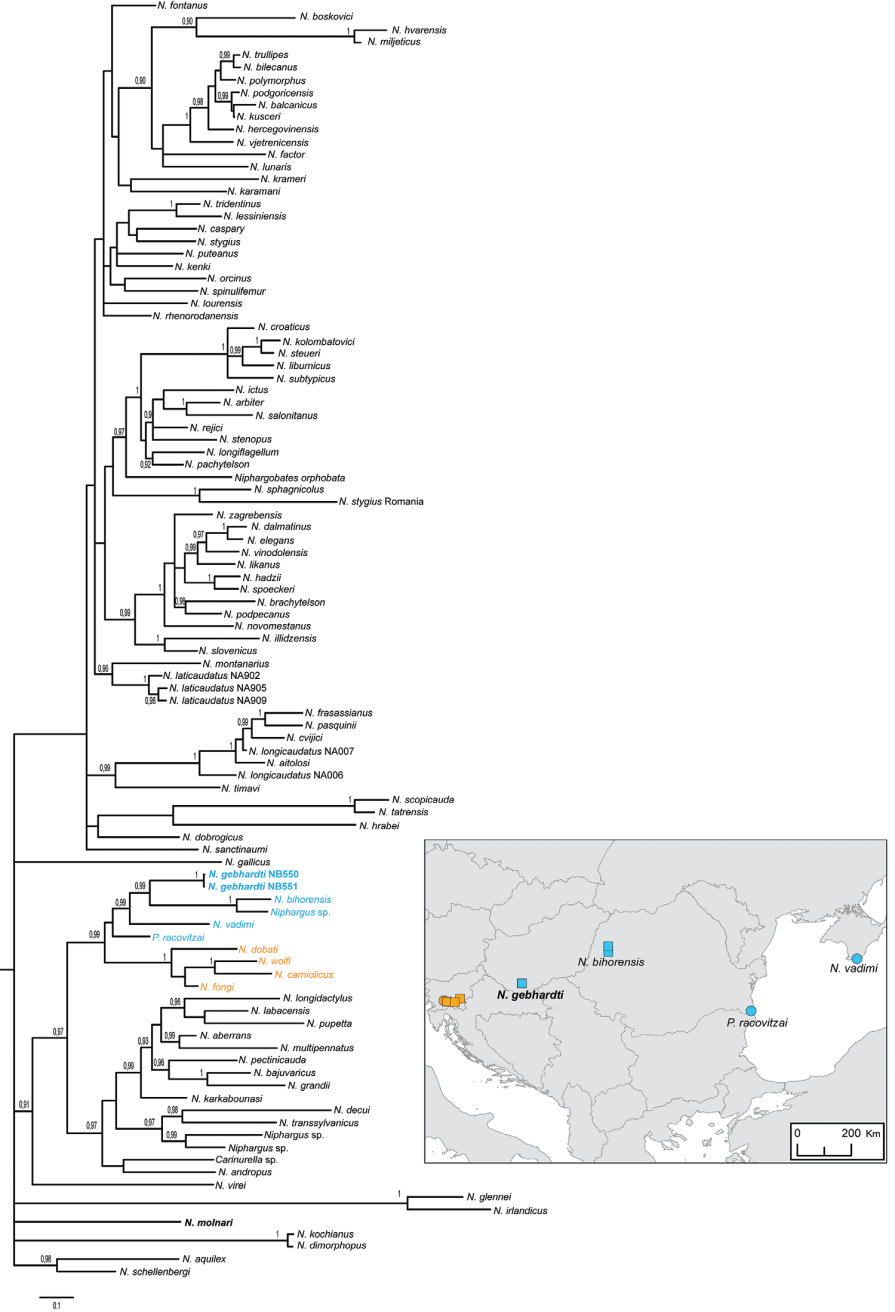
Bayesian phylogenetic tree of 104 amphipod taxa (including *Niphargus molnari* and *Niphargus gebhardti*) based on COI, 28S and histone (H3) sequences. Map represents distribution of the clade with *Niphargus gebharti*. Squeres represent epikarstic species and circles species from other subterranean habitats.

Phylogenetic relationships within the genus *Niphargus* (Fig. 17) showed that the two redescribed species of *Niphargus* from Hungary are not phylogenetically closely related. Phylogenetic relationship of *Niphargus molnari* to the rest of *Niphargus* species is unclear; species is nested within basal polytomy. *Niphargus gebhardti* belongs to the clade of Central to Eastern European species. The focal species is in sister relationship with a pair of morphologically cryptic species endemic to Western Carpathian (*Niphargus bihorensis*, see Meleg et al. 2013). Other closely related species include *Niphargus vadimi* from Crimea, *Pontoniphargus racovitzai* from Eastern Romania and a clade of epikarstic and interstitial species from Southern Slovenia (*Niphargus fongi*, *Niphargus carniolicus*, *Niphargus wolfi* and *Niphargus dobati*).

### Remarks on ecology and distribution

**Figure 18.**
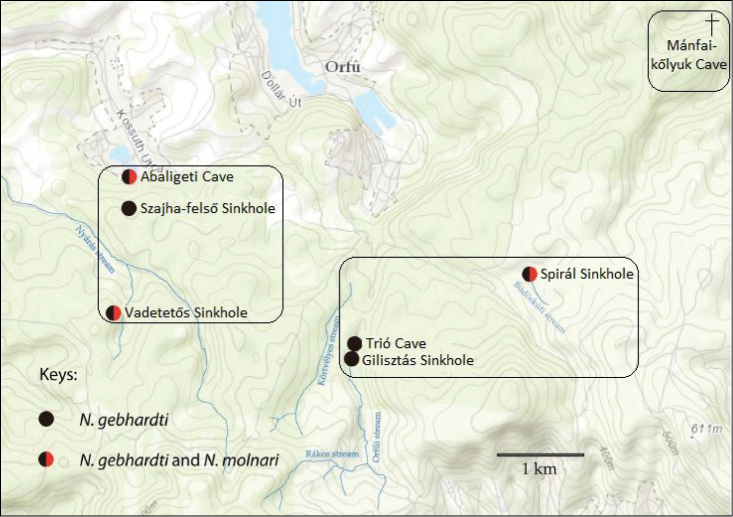
Distribution of *Niphargus molnari* and *Niphargus gebhardti* within the Western Mecsek.

**Figure 19.**
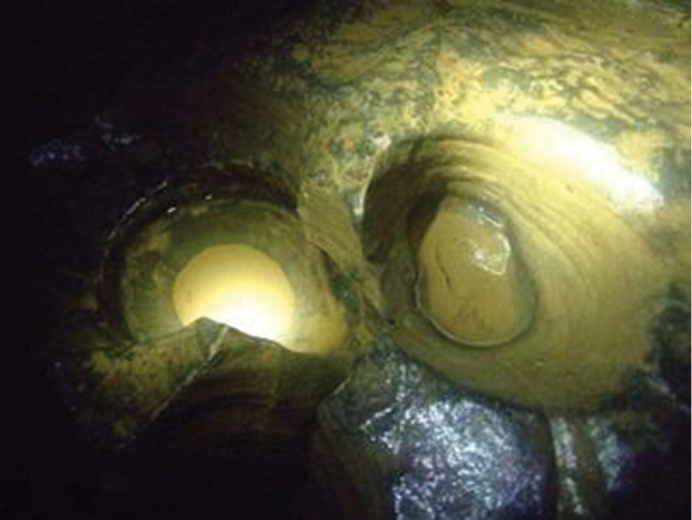
Small pool formed by dripping water, one of the occupied microhabitats of *Niphargus gebhardti* in the Trió Cave.

Among the studied two species, *Niphargus gebhardti* was collected more frequently, as it was found in five other caves of the Western Mecsek in addition to the type locality, namely Trió Cave, Gilisztás Sinkhole, Szajha-felső Sinkhole, Vadetetős Sinkhole and Spirál Sinkhole (Fig. 18). In most of these, two types of water bodies exist: i) small pools of residual- or percolated/dripping water and ii) streams or minor streaming water. Amount of water in the caves is dependent on the rainfall in the surface. In all six caves, *Niphargus gebhardti* specimens were found in isolated, shallow pools in limestone, sinter or clay, most likely formed by dripping water (Fig. 19). Specimens were never observed in streams or any other streaming waters. During our repeated visits between 2010 and 2013 (altogether 24 visits in the 6 caves), the same pools were checked every time and some specimens were always found in them (except when the pools dried out). Once it was observed that a group of *Niphargus gebhardti* (approximately 20 specimens) were fed upon a dead *Oxychilus* snail in a pool.

*Niphargus molnari* was observed in the Abaligeti Cave and in two sinkholes that the other species (*Niphargus gebhardti*) was also inhabited, Spirál Sinkhole and Vadetetős Sinkhole (Fig. 18). Density of *Niphargus molnari* was high in the stream of the Western 2 collateral of the Abaligeti Cave, however in the other two caves only a few specimens were found in streaming water, always in deeper parts of the caves. The two species were always spatially well segregated. In the Abaligeti Cave *Niphargus molnari* coexisted with *Protelsonia hungarica* Méhely, 1924 (endemic aquatic troglobiont isopod of the cave) and with the troglomorph specimens of *Gammarus fossarum* Koch, 1836.

## Discussion

Due to its protected geographical situation, since the Tertiary, the area of Mecsek may have played a refugial role during the alternating warmer and colder eras, preserving old lineages of Crustaceans. They presumably ensconced into subterranean aquatic habitats from searing creaks of the Paratethys Sea, that encompassed the islands of the Mecsek. Then, by degress, they had been adapted to the subterranean conditions in both physiological and morphological features (Méhely 1925). According to results of our phylogenetic analysis, *Niphargus molnari* and *Niphargus gebhardti* represent completely distinct lineages, which colonized the Mecsek area independently. The two species are spatially segregated within the same caves. *Niphargus gebhardti* inhabits isolated pools of stagnant water, which fed by percolating water from the limestone fissures, so called epikarst. Interestingly *Niphargus gebhardti* is apparently phylogenetically related to epikarstic species from Slovenia. On the contrary, *Niphargus molnari* was always found in streaming waters.

The distribution range of the two endemic species is small, the most distant caves are seven kilometers far. These caves belong to three different catchment areas (Fig. 18). Despite of our repeated visits and careful searching, *Niphargus* specimens were not found in the Mánfai-kőlyuk Cave. *Niphargus molnari* supposedly has gone extinct in its type locality as it is ruined due to the industrial utilization of the cave (Angyal 2012). Moreover, the type locality of *Niphargus gebhardti* – which is a touristic cave with 80.000 annual visitors – may be also endangered. Considering the extremely narrow distributional range of the two species and the vulnerability of their populations, *Niphargus molnari* and *Niphargus gebhardti* are suggested to be placed into the ’Vulnerable (VU)’ category according to the following criteria of IUCN Red List of Threatened Species (IUCN 2012): i) number of locations is ≤ 10 (‘B2’) and ii) area of occupancy is less than 20 km^2^ (‘D2’).’

Hidrologically connected caves are in quadrats.

## Conclusions

Some highly endemic, troglobiont invertebrate taxa are known from the Southern Hungarian Mecsek Mts. Two of them, the blind amphipod *Niphargus molnari* Méhely, 1927 and *Niphargus gebhardti* Schellenberg, 1934 have been rediscribed, applying the modern approach of integrative taxonomy. Comparative scanning electron microscopy used for first time on niphargids, and it proved to be a rather useful method in analysing and illustrating of barely visible diagnostic characters. As contributions to the future molecular genetic studies on niphargids, cytochrome c oxidase subunit I (COI) sequences as barcodes of *Niphargus molnari* and *Niphargus gebhardti* are now available for the public. The phylogenetic analyses have shown that the two species – which are spatially segregated in caves where they coexist – represent completely distinct lineages and may have colonized the Mecsek area independently. Phylogenetic relationship of *Niphargus molnari* to the rest of *Niphargus* species is for the present not clear. *Niphargus gebhardti* is closely related to a clade of epikarstic species from Southern Slovenia and to cryptic species endemic to Western Carpathians. New localities of both species have been found. The two species are suggested for legal protection, they should be listed into ‘Vulnerable’ category of the IUCN Red List of Threatened Species.

## Supplementary Material

XML Treatment for Niphargus molnari

XML Treatment for Niphargus gebhardti

## Supplementary materials

Supplementary material 1 Protocols and thermo profiles used in molecular studies Data type: Primers, molecular protocols This dataset is made available under the Open Database License (http://opendatacommons.org/licenses/odbl/1.0/). The Open Database License (ODbL) is a license agreement intended to allow users to freely share, modify, and use this Dataset while maintaining this same freedom for others, provided that the original source and author(s) are credited.Dorottya Angyal, Gergely Balázs, Valerija Zakšek, Virág Krízsik, Cene Fišer

Supplementary material 2 List of taxa and sequence data used in phylogenetic analysis Data type: Sequence data This dataset is made available under the Open Database License (http://opendatacommons.org/licenses/odbl/1.0/). The Open Database License (ODbL) is a license agreement intended to allow users to freely share, modify, and use this Dataset while maintaining this same freedom for others, provided that the original source and author(s) are credited.Dorottya Angyal, Gergely Balázs, Valerija Zakšek, Virág Krízsik, Cene Fišer

Supplementary material 3 Original descriptions of *Niphargus molnari* and *Niphargus gebhardti* Data type: Descriptions This dataset is made available under the Open Database License (http://opendatacommons.org/licenses/odbl/1.0/). The Open Database License (ODbL) is a license agreement intended to allow users to freely share, modify, and use this Dataset while maintaining this same freedom for others, provided that the original source and author(s) are credited.Dorottya Angyal, Gergely Balázs, Valerija Zakšek, Virág Krízsik, Cene Fišer

## References

[B1] Angyal D (2012) Invertebrate fauna of the Mánfai-kőlyuk Cave (Mecsek, SW Hungary) in the light of utilization by waterworks – previous results. Természetvédelmi Közlemények 18: 24–33. [In Hungarian]

[B2] Angyal D Balázs G (2013) Distinguishing characters of Niphargus gebhardti Schellenberg, 1934 and Niphargus molnari Méhely, 1927 (Crustacea: Amphipoda): a clarification. Opuscula Zoologica, Budapest 44(1): 3–8.

[B3] Birstein JA (1961) Subterranean Amphipodes of the Crimea. Biospeologica Sovietica. XIV. Boljeten Moskovskogo občestva ispitateljev prirodi 66(6): 126–144.

[B4] Colgan DJ Ponder WF Eggler PE (2000) Gastropod evolutionary rates and phylogenetic relationships assessed using partial 28S rDNA and histone H3 sequences. Zoologica Scripta 29(1): 29–63.

[B5] Dezső J (2011) A dél-dunántúli karsztos területek morfológiai, üledékföldtani vizsgálata és összehasonlító értékelésük analóg képződményekkel. PhD thesis, University of Pécs, Pécs, Hungary [In Hungarian]

[B6] Dudich E (1941) Die im Gebiete des historischen Ungarn nachgewiesenen Amphipoden. Fragmenta Faunistica Hungarica 4(1–4): 14–20. [In German]

[B7] Fišer C Zagmajster M (2009) Cryptic species from cryptic space: the case of Niphargus fongi sp. n. (Amphipoda, Niphargidae). Crustaceana 82(5): 593–614.

[B8] Fišer C Coleman CO Zagmajster M Zwittnig B Gerecke R Sket B (2010) Old museum samples and recent taxonomy: a taxonomic, biogeographic and conservation perspective of the Niphargus tatrensis species complex (Crustacea: Amphipoda). Organisms Diversity & Evolution 10: 5–22.

[B9] Fišer C Trontelj P Luštrik R Sket B (2009) Towards a unified taxonomy of Niphargus (Crustacea: Amphipoda): a review of morphological variability. Zootaxa 2061: 1–22.

[B10] Folmer O Black M Hoeh W Lutz R Vrijenhoek R (1994) DNA primers for amplification of mitochondrial cytochrome c oxidase subunit I from diverse metazoan invertebrates. Molecular Marine Biology and Biotechnology 3: 294–299. 7881515

[B11] Gebhardt A (1933) Vergleichung der Tierwelt der Abaligeter – und Mánfaer Höhlen. Állattani Közlemények 30(1–2): 36–44. [In Hungarian and German]

[B12] Gebhardt A (1934) Az Abaligeti barlang élővilága. Mathematikai és Temészettudományi Közlemények 37: 1–264. [In Hungarian]

[B13] Gebhardt A (1963) A Mecsek hegység barlangjainak biológiai vizsgálata. Janus Pannonius Múzeum Évkönyve 8: 5–32. [In Hungarian]

[B14] Gebhardt A (1967) A Mecsek hegység állatvilágának térbeli elterjedése élőhelyek szerint. Janus Pannonius Múzeum Évkönyve 12: 7–14. [In Hungarian]

[B15] Hou Z Sket B Li S (2013) Phylogenetic analyses of Gammaridae crustacean reveal different diversification patterns among sister lineages in the Tethyan region. Cladistics 30: 352–365. 10.1111/cla.12055 34794244

[B16] IUCN (2012) IUCN Red List Categories and Criteria: Version 3.1. Second edition IUCN, Gland, Switzerland and Cambridge, UK, iv + 32 pp. doi: 10.1111/cla.12055

[B17] Karaman S (1932) 5. Beitrag zur Kenntnis der Süsswasser-Amphipoden (Amphipoden unterirdischer Gewässer). Prirodoslovne razprave, Ljubljana 2: 179–232. [In German]

[B18] Karaman GS (1980) First discovery of Niphargus bihorensis Schell. 1940 (fam. Gammaridae) in Italy with remarks to N elegans Garb. 1894. (Contribution to the knowledge of the Amphipoda 111). Glas. Repub. Zavoda Zašt. Prir.- Prir. Muz. Titograd 13: 71–80.

[B19] Karaman GS Ruffo S (1986) Amphipoda: *Niphargus* – group (Niphargidae sensu Bousfield, 1982). In: Botosaneau L (Ed.) Stygofauna mundi, a faunistic, distributional, and ecological synthesis of the world fauna inhabiting subterranean waters (including marine interstitial), 514–534.

[B20] Karaman G (1989) The redescription of *Niphargus carniolicus* Sket 1960 (fam. Niphargidae) with remarks to its new taxonomic position (Contribution to the knowledge of the Amphipoda 195). Poljepriveda i Šumarstvo 35: 13–28.

[B21] Katoh K Standley DM (2013) MAFFT multiple sequence alignment software version 7: improvements in performance and usability. Molecular Biology and Evolution 30: 772–780. 23329690 10.1093/molbev/mst010 PMC3603318

[B22] Keane TM Creevey CJ Pentony MM Naughton TJ McInerney JO (2006) Assessment of methods for amino acid matrix selection and their use on empirical data shows that ad hoc assumptions for choice of matrix are not justified. BMC Evolutionary Biology 6: . doi: 10.1186/1471-2148-6-29 10.1186/1471-2148-6-29 PMC1435933 16563161

[B23] Mamos T Wattier R Majda A Sket B Grabowski M (2014) Morphological vs. molecular delineation of taxa across montane regions in Europe: The case study of *Gammarus balcanicus* Schäferna, 1922 (Crustacea: Amphipoda). Journal of Zoological Systematics & Evolutionary Research 52(3): 237–248.

[B24] Meleg IN Zakšek V Fišer C Kelemen BS Moldovan OT (2013) Can environment predict cryptic diversity? The case of *Niphargus* inhabiting Western Carpathian groundwater. PLoS ONE 8(10): . doi: 10.1371/journal.pone.0076760 10.1371/journal.pone.0076760 PMC3804523 24204671

[B25] Méhely L (1925) Ein Lebendes Fossil. (Protelsonia hungarica, nov. gen., n. sp., Ein blinder Isopode aus Ungarn.) Matematikai és Természettudományi Értesítő 41: 185–192. [In Hungarian and German]

[B26] Méhely L (1927) Új férgek és rákok a magyar faunában. Neue Würmer und Krebse aus Ungarn, Budapest 1927: 1–19. [In Hungarian and German]

[B27] Méhely L (1937) Niphargus hungaricus ein neuer Amphipode aus Ungarn. Zoologischer Anzeiger 120: 117–119.

[B28] Méhely L (1941) A Niphargus-kutatás új útjai. Neue Wege der Niphargus-Forschung. Budapest 1941: 1–36. [In Hungarian and German]

[B29] Miller MA Holder MT Vos R Midford PE Liebowitz T Chan L Hoover P Warnow T (2012) The CIPRES portals CIPRES. http://www.phylo.org/sub_sections/portal

[B30] Padial JM Miralles A De la Riva I Vences M (2010) The integrative future of taxonomy. Frontiers in Zoology 7: . doi: 10.1186/1742-9994-7-16 10.1186/1742-9994-7-16 PMC2890416 20500846

[B31] Ronquist F Huelsenbeck JP (2003) MrBayes 3: Bayesian phylogenetic inference under mixed models. Bioinformatics 19: 1572–1574. 12912839 10.1093/bioinformatics/btg180

[B32] Schellenberg A (1933) Weitere deutsche und ausländische Niphargiden. Zoologischer Anzeiger 102: 22–23. [In German]

[B33] Schellenberg A (1934) Amphipoden aus Quellen, Seen und Höhlen. Zoologischer Anzeiger 106: 200–209. [In German]

[B34] Schellenberg A (1935) Schlüssel der Amphipodengattung Niphargus mit Fundortangaben und mehreren neuen Formen. Zoologischer Anzeiger 111: 204–211. [In German]

[B35] Sket B (1960) Einige neue Formen der Malacostraca aus Jugoslawien III. Bulletin Scientifique 5(5): 73–75. [In German]

[B36] Sket B (1999) Niphargus aquilex dobati ssp. n. (Crustacea) from the karst of Slovenia. Mitteilungen des Verbandes der deutschen Höhlen- und Karstforscher 45(2): 54–56.

[B37] Švara V Delić T Rada T Fišer C (submitted) Molecular phylogeny of Niphargus boskovici (Crustacea: Amphipoda) reveals a new species from epikarst. 10.11646/zootaxa.3994.3.2 26250278

[B38] Verovnik R Sket B Trontelj P (2005) The colonization of Europe by the freshwater crustacean Asellus aquaticus (Crustacea: Isopoda) proceeded from ancient refugia and was directed by habitat connectivity. Molecular Ecology 14: 4355–4369. 16313598 10.1111/j.1365-294X.2005.02745.x

[B39] Zakšek V Sket B Trontelj P (2007) Phylogeny of the cave shrimp Troglocaris: evidence of a young connection between Balkans and Caucasus. Molecular Phylogenetics and Evolution 42: 223–235. 16935529 10.1016/j.ympev.2006.07.009

