# Supplementary material for: Redescription of two subterranean amphipods Niphargus molnari Méhely, 1927 and Niphargus gebhardti Schellenberg, 1934 (Amphipoda, Niphargidae) and their phylogenetic position
Source: Zookeys. 2015 Jun 24;(509):53–85. doi: 10.3897/zookeys.509.9820 (PMC4493343; doi:10.3897/zookeys.509.9820)
Supplement: Supplementary material 1 — Protocols and thermo profiles used in molecular studies [file zookeys-509-053-s001.doc]

**Supplementary file 1.** Protocols and thermo profiles used in molecular studies

1. COI

*N. gebhardti*

Primers: F: LCO 1490, R: HCO 2198

PCR reactions (25 µl) were obtained by mixing 13,85 µl mQ water, 2,5 µl 10X PCR buffer, 2,5 µl dNTP mix (2mM), 1,5 µl of each primers (5µM), 0,15 µl Fermentas Dream Taq® (5U/ µl) and 3 µl DNA extract. PCR temperature conditions were as follows: initial denaturation for 3 min at 94°C, denaturation for 45 sec at 94°C, hybridization for 45 sec at 48°C, and polymerization for 1 min at 72°C. After thirty cycles a final extension for 3 min at 72°C was added.

*N. molnari*

Primers: F: LCO 1490, R: HCO 2198

PCR reactions (15 µl) were obtained by mixing 11 µl mQ water, 1,5 µl 10X PCR buffer (with MgCl_2_) , 1,5 µl dNTP, 0,2 µl of each primers (5µM), 0,07 µl BIOTOOLS DNA Polymerase® (5U/ µl) and 1 µl DNA extract. PCR temperature conditions were as follows: initial denaturation for 4 min at 95°C, denaturation for 1 min at 95°C, hybridization for 1 min at 45°C, and polymerization for 2 min 30 sec at 72°C. After fourty cycles a final extension for 7 min at 72°C was added.

1. 28S rDNA

*N. gebhardti, N. molnari*

Primers: F: 28S lev2, R: 28S des2, 28S rtest2

PCR reactions (15 µl) were obtained by mixing 11 µl mQ water, 1,5 µl 10X PCR buffer (with MgCl_2_) , 1,5 µl dNTP, 0,2 µl of each primers (5µM), 0,07 µl BIOTOOLS DNA Polymerase® (5U/ µl) and 1 µl DNA extract. PCR temperature conditions were as follows: initial denaturation for 3 min at 94°C, denaturation for 30 sec at 94°C, hybridization for 1 min at 45°C, and polymerization for 1 min at 72°C. After fourty cycles a final extension for 5 min at 72°C was added.

1. H3

*N. gebhardti, N. molnari*

Primers: F: H3aF2, R: H3aR2

PCR reactions (15 µl) were obtained by mixing 11 µl mQ water, 1,5 µl 10X PCR buffer (with MgCl_2_) , 1,5 µl dNTP, 0,2 µl of each primers (5µM), 0,07 µl BIOTOOLS DNA Polymerase® (5U/ µl) and 1 µl DNA extract. PCR temperature conditions were as follows: initial denaturation for 3 min at 94°C, denaturation for 45 sec at 94°C, hybridization for 1 min at 46°C, and polymerization for 1 min at 72°C. After fourty cycles a final extension for 3 min at 72°C was added.

| **Marker** | **Primer** | **Direction** | **Sequence** | **Reference** |
| --- | --- | --- | --- | --- |
| COI | LCO 1490 | Forward | 5’ GGTCAACAAATCATAAAGATATTGG 3’ | Folmer et al., 1994 |
| COI | HCO 2198 | Reverse | 5’ TAAACTTCAGGGTGACCAAAAAAT 3’ | Folmer et al., 1994 |
| 28S rDNA | 28S lev2 | Forward | 5’ CAAGTACCGGTGAGGGAAAGTT 3’ | Verovnik et al., 2005 |
| 28S rDNA | 28S des2 | Reverse | 5’ GTTCACCATCTTTCGGGTC 3’ | Zakšek et al., 2007 |
| 28S rDNA | 28S rtest2 | Reverse | 5’ AGGGAAACTTCGGA-GGGAACC 3’ | Verovnik et al., 2005 |
| H3 | H3aF2 | Forward | 5’ ATGGCTCGGTACCAAGCAGAC 3’ | Colgan et al., 2000 |
| H3 | H3aR2 | Reverse | 5’ ATTTCCTTGGGCATGATTGTTAC 3’ | Colgan et al., 2000 |
