# Supplementary material for: Redescription of two subterranean amphipods Niphargus molnari Méhely, 1927 and Niphargus gebhardti Schellenberg, 1934 (Amphipoda, Niphargidae) and their phylogenetic position
Source: Zookeys. 2015 Jun 24;(509):53–85. doi: 10.3897/zookeys.509.9820 (PMC4493343; doi:10.3897/zookeys.509.9820)
Supplement: Supplementary material 2 — List of taxa and sequence data used in phylogenetic analysis [file zookeys-509-053-s002.doc]

**Supplementary file 2**. List of taxa and sequence data used in phylogenetic analysis.

| **Species** | **28S rDNA** | **Histone H3** | **COI** | **Locality** |  |
| --- | --- | --- | --- | --- | --- |
| *Niphargus gebhardti* | KP967556  (NB550) | KP967550  (NB550) | KP967553  (NC041) | Abaligeti cave |  |
| *Niphargus gebhardti* | KP967557  (NB551) | KP967551  (NB551) | KP967554  (NC037) | Szajha-felső sinkhole |  |
| *Niphargus molnari* | KP967555 | KP967549 | KP967552 | Abaligeti cave; NB555 |  |
| *Carinurella paradoxa* | - | KR905901 | KR905829 | Torre, Ruda, Monfalcone, ITA; NA738 |  |
| *Pontoniphargus racovitzai* | KF290023 | - | - | Pestera de la Movile, Mangalia, Dobrogea, ROM |  |
| *Niphargus dolichopus* | EU693297 | JQ815490 | KT007331 | Suvaja pećina, Lušci palanka, Sanski most, BIH; NA076 |  |
| *Niphargus fontanus* | EF617304 | - | KC315635 | Little stour river, Littlebourn, Littlebourn, / Amersham, Buckinghamshire, BG |  |
| *Niphargus frasassianus* | GU973411 | - | GU973034 | Grotte di Frasaasi, Fabriano, Perugia, IT |  |
| *Niphargus gallicus* | KF290033 | - | KF290225 | str. Bisericii 39, Dulcești, Mangalia, ROM |  |
| *Niphargus glennei* | KC315617 | - | KC315644 | Plympton farm catchpit, Plymouth, Devon, GB |  |
| *Niphargus ictus* | GU973415 | - | GU973012 | Grotte di Frasaasi, Fabriano, Perugia, IT |  |
| *Niphargus irlandicus* | KC315618 | - | KC315647 | Carrigacrump Quarry, Coyne, Cork, GB |  |
| *Niphargus kolombatovici* | JQ815553 | JQ815522 | - | Žira, Turkovići, Ravno, BIH; NA964 |  |
| *Niphargus liburnicus* | KT007478 | - | KT007418 | Grotta Andrea, Iamiano, Doberdob, IT, NB018 |  |
| *Niphargus montanarius* | GU973419 | - | GU973003 | Grotte di Frasaasi, Fabriano, Perugia, IT |  |
| *Niphargus steueri* | JQ815551 | JQ815519 | KT007358 | Jama pod krogom, Sočerga, Koper, SI |  |
| *Synurella ambulans* | EF617236 | - | KR905770 | forest ditch near Dept. Of. Biology, Ljubljana, Ljubljana, SI, NA002 |  |
| *Niphargus virei* | EF617237 | JQ815467 | KR905771 | Dorpstraat 7 (well), Reijmerstok, Limburg, NED, NA003 |  |
| *Niphargus longicaudatus [Cres]* | EF617240 | KJ566705 | KR905772 | Retec (source), Lubenice, Island of Cres, HR, NA006 |  |
| *Niphargus longicaudatus [Gragnano]* | EF617241 | JQ815469 | - | Stream near the road Monte Faito-Vico Equense, Casola, Napoli, IT; NA007 |  |
| *Niphargus pasquinii* | EF617244 | JQ815471 | KR905773 | Sorgenti San Vittorino, San Vittorino, Castel Sant'Angelo, IT; NA010 |  |
| *Niphargus dobati* | EF617247 | JQ815499 | KR905774 | Rakov Škocjan, Zelše, Cerknica, SI; NA013 |  |
| *Niphargus wolfi* | EF617250 | JQ815500 | KR905775 | Križna jama, Bločice, Lož, SI; NA015 |  |
| *Niphargus carniolicus* | EF617252 | JQ815501 | KR905776 | Jama pod gradom Luknja, Prečna, Novo mesto, SI; NA017 |  |
| *Niphargus fongi* | EF617253 | JQ815472 | - | Dolga jama pri Koblarjih, Koblarji, Kočevje, SI; NA018 |  |
| *Niphargus longidactylus* | EF617256 | JQ815473 | - | Sneberje (Sava freatic waters), Ljubljana, Ljubljana, SI; NA021 |  |
| *Niphargus labacensis* | EF617257 | JQ815474 | KR905777 | Tomačevo (intersticial waters), Ljubljana, Ljubljana, SI; NA022 |  |
| *Niphargus pectinicauda* | EF617258 | JQ815475 | KR905778 | Tomačevo (intersticial waters), Ljubljana, Ljubljana, SI; NA023 |  |
| *Niphargus bajuvaricus* | EF617259 | JQ815476 | KT027378 | Well A96, Lobau, Wien, AUT; NA024 |  |
| *Niphargus aberrans* | EF617260 | - | - | Planinska jama, Kačja vas, Planina, SI; NA025 |  |
| *Niphargus scopicauda* | EF617261 | JQ815477 | KR905779 | Huda luknja pri Gornjem Doliču, Završe, Slovenj Gradec, SI; NA026 |  |
| *Niphargus tatrensis* | EF617263 | - | - | Lodowe zrodlo (Icy spring), POL; NA028 |  |
| *Niphargus aquilex* | EF617264 | KP300936 | KC315626 | Marden, Marden, Wes Sussex, GB; NA029 |  |
| *Niphargus schellenbergi* | EF617267 | JQ815478 | KR905780 | Well near road 800 m SE from Heyd, Heyd, Durbuy, BEL; NA032 |  |
| *Niphargus sphagnicolus* | EF617270 | - | KR858495 | Mostec, Rožnik, Ljubljana, SI; NA035 |  |
| *Niphargus boskovici* | EF617271 | JQ815502 | KR905781 | Bjelušica, Zavala, Popovo polje, BIH; NA036 |  |
| *Niphargus hvarensis* | EF617273 | JQ815479 | KR905782 | Trsteno, Dubrovnik, Dubrovnik, HR; NA038 |  |
| *Niphargus krameri* | EF617275 | JQ815503 | - | Fojba, Šestani, Pazin, HR; NA040 |  |
| *Niphargus trullipes* | EF617281 | JQ815504 | KR905783 | Vjetrenica, Zavala, Popovo polje, BIH; NA046 |  |
| *Niphargus polymorphus* | EF617282 | JQ815505 | KR905784 | Obodska pećina, Rijeka Crnojevića, Cetinje, MNE; NA047 |  |
| *Niphargus rejici* | EF617283 | JQ815481 | KR905785 | Podpeško jezero, Jezero, Ig, SI; NA048 |  |
| *Niphargus stenopus* | EF617284 | JQ815506 | - | Jama pod gradom Luknja, Prečna, Novo mesto, SI; NA049 |  |
| *Niphargus arbiter [Krk]* | EF617286 | KR905885 | KR905786 | Spring in port Vrbnik, Vrbnik, Krk, CRO; NA050 |  |
| *Niphargus salonitanus* | EF617289 | JQ815483 | KR905788 | Gospa od Stomorije spring, Kaštel Stari, Split, HR; NA053 |  |
| *Niphargus zagrebensis [Gadina]* | EF617295 | KR905886 | KR905789 | Gadina, Loka, Črnomelj, SI; NA059 |  |
| *Niphargus dalmatinus* | EF617296 | JQ815484 | KR905790 | Biba spring, Vrana, Pakoštane, HR; NA060 |  |
| *Niphargus elegans* | EF617297 | JQ815485 | KR905791 | San Pancrazio, San Pancrazio, Verona, IT; NA061 |  |
| *Niphargus vinodolensis* | EF617298 | JQ815486 | KR905792 | Ceovići, Bačići, Novi Vinodolski, HR; NA062 |  |
| *Niphargus tridentinus* | EF617299 | JQ815487 | KR905793 | Grotta Bus Pursi, Lumezzane, Brescia, IT; NA063 |  |
| *Niphargus lessiniensis* | EF617300 | JQ815488 | - | Grotta del Aqua, Ponte de Veja, Monte Lessini, IT; NA064 |  |
| *Niphargus puteanus* | EF617302 | KJ566709 | KR905795 | Gasthof Zur Walba, Pentling, Pentling, GER; NA066 |  |
| *Niphargus brachytelson* | EU693293 | JQ815489 | KR905797 | Lukova jama pri Zdihovem, Suhor, Kočevje, SI; NA071 |  |
| *Niphargus caspary* | EU693291 | KJ566712 | - | Tuebingen, Tuebingen, Tuebingen: GER; NA073 |  |
| *Niphargus factor* | EU693298 | JQ815508 | KR905798 | Vjetrenica, Zavala, Popovo polje, BIH; NA078 |  |
| *Niphargus grandii* | EU693300 | KJ566715 | KR905799 | Torre, Ruda, Monfalcone, IT; NA080 |  |
| *Niphargus longicaudatus [Lisimachia]* | EU693310 | KP133142 | - | Lisimachia, Klisorevmata, Agrinio, GRE; NA081 |  |
| *Niphargus hadzii* | EU693301 | KR905887 | KR905800 | Izvir pod orehom, Verd, Vrhnika, SI; NA082 |  |
| *Niphargus hrabei* | EU693302 | KJ566716 | KR905801 | Stream near the road W from Lupoglav, Lupoglav, Zagreb, HR; NA083 |  |
| *Niphargus illidzensis* | EU693304 | JQ815491 | KR905802 | Vrelo Bosne, Ilidža, Sarajevo, BIH; NA084 |  |
| *Niphargus karamani* | EU693305 | KR905888 | KR905803 | Fram 119 (well), Fram, Maribor, SI; NA085 |  |
| *Niphargus kenki* | KR905869 | - | KR905804 | Spring near to Sodna vas 25, Sodna vas, Podčetrtek, SI; NA086 |  |
| *Niphargus kochianus* | EU693308 | JQ815492 | - | Saint Albans, Hertfordshire, Hertfordshire, GB, NA090 |  |
| *Niphargus longiflagellum* | EU693311 | JQ815520 | KR905805 | Podpeška jama, Videm, Grosuplje, SI; NA093 |  |
| *Niphargus lourensis* | EU693312 | - | KR905806 | Louros spring, Vouliasta, Ioannina, GRE; NA094 |  |
| *Niphargus lunaris* | EU693313 | KR905889 | KR905807 | Bubanj vrelo, Dolac Donji, Trilj, HR; NA095 |  |
| *Niphargus novomestanus* | EU693314 | JQ815509 | KR858496 | Tominčev studenec, Žužemberk, Žužemberk, SI; NA096 |  |
| *Niphargus orcinus* | EU693315 | JQ815510 | KR905808 | Križna jama, Bločice, Lož, SI; NA099 |  |
| *Niphargus pachytelson* | EU693316 | JQ815511 | KR905809 | Podpeška jama, Videm, Grosuplje, SI; NA100 |  |
| *Niphargus podpecanus* | EU693317 | JQ815512 | KR905810 | Podpeška jama, Videm, Grosuplje, SI; NA101 |  |
| *Niphargus pupetta* | EU693318 | KJ566717 | - | Tomačevo (intersticial waters), Ljubljana, Ljubljana, SI; NA102 |  |
| *Niphargus rhenorodanensis* | EU693319 | KJ566719 | KR905811 | Grotte Cormoran, Torcieu, Lyon, FR; NA104 |  |
| *Niphargus sanctinaumi* | EU693320 | KP133144 | KR905812 | Sveti Naum spring, Sv. Naum, Ohrid, MAC; NA105 |  |
| *Niphargus slovenicus* | EU693322 | JQ815493 | KR905813 | Stražišče, Kranj, Kranj, SI; NA106 |  |
| *Niphargus spinulifemur* | EU693323 | JQ815494 | KR858500 | Stream NE to Hrastovlje, Hrastovlje, Koper, SI; NA107 |  |
| *Niphargus spoeckeri* | EU693324 | JQ815513 | KR905814 | Pivka jama, Veliki otok, Postojna, SI; NA108 |  |
| *Niphargus stygius* | KR905870 | KR905890 | KR905815 | Jelenska jama, Borovnica, Vrhnika, SI; NA110 |  |
| *Niphargus subtypicus* | EU693326 | JQ815514 | KT007433 | Jama pod gradom Luknja, Prečna, Novo mesto, SI; NA112 |  |
| *Niphargus timavi* | EU693327 | JQ815495 | KR858497 | Labodnica, Trebiciano, Trieste, IT; NA114 |  |
| *Niphargus vjetrenicensis* | EU693329 | JQ815521 | KR858499 | Vjetrenica, Zavala, Popovo polje, BIH; NA116 |  |
| *Niphargus dimorphopus* | EU693296 | JQ815496 | - | Gulpen, Gulpen, Limburg, NED; NA125 |  |
| *Niphargus dobrogicus* | KR905871 | KR905891 | KR905816 | Well N to Limanu, Mangalia, Dobrogea, ROM; NA140 |  |
| *Niphargus vadimi* | KR905872 | KR905892 | KR905817 | Skelska peščera, Rodnikovo, Krym, UKR; NA144 |  |
| *Niphargus cvijici* | JQ815554 | JQ815516 | KR905819 | Popovo polje, Ravno, Ravno, BIH; NA147 |  |
| *Niphargus hercegovinensis* | JQ815549 | JQ815517 | KR905820 | Žira, Turkovići, Ravno, BIH; NA151 |  |
| *Niphargus stygius [Romania]* | KJ566693 | KJ566720 | KR905821 | Valeni (wells), Ploiesti, Prahova, ROM; NA152 |  |
| *Niphargus decui* | KF719272 | KR905894 | KR905822 | Limanu springs, Mangalia, Dobrogea, ROM; NA154 |  |
| *Niphargus podgoricensis* | KR905875 | KR905896 | KR905824 | Spring at Dobro polje, Dobro polje, Podgorica, MNE; NA166 |  |
| *Niphargus multipennatus* | KJ566700 | KJ566721 | KR905825 | Tomačevo (intersticial waters), Ljubljana, Ljubljana, SI; NA169 |  |
| *Niphargus bilecanus* | JQ815550 | - | KR905826 | Ljelješnica, Poraslica, Dabarsko polje, BIH; NA182 |  |
| *Niphargus karkabounasi* | KR905877 | KR905898 | - | Agios Theodoridi, Agios Theodoridi, Korinthos, GRE; NA217 |  |
| *Niphargus miljeticus* | KR905878 | KR905899 | - | Vodice, Babino polje, island of Mljet, HR; NA500 |  |
| *Niphargus likanus* | JQ815441 | JQ815498 | KR905828 | Jama v kamnolomu, Vinica, Črnomelj, SLO; NA523 |  |
| *Niphargobates orophobata* | KR905879 | KR905900 | - | Planinska jama, Planina, SI; NA546 |  |
| *Niphargus balcanicus* | EF617280 | JQ815507 | KR905796 | Vjetrenica, Zavala, Popovo polje, BIH; NA070 |  |
| *Niphargus kusceri* | JQ815443 | KR905929 | KR905767 | Obodska pećina, Rijeka Crnojevića, Cetinje, / Njegoševa pećina, Njeguši, Kotor, MNE; NB422 |  |
| *Niphargus bihorensis* | KF218727 | KF218657 | - | Meziad cave; Meziad, Pădurea Craiului, ROM; NA792 |  |
| *Niphargus sp. 4* | KF218731 | KF218731 | KF218667 | Vadu cave; Pădurea Craiului, ROM; NA794 |  |
| *Niphargus laticaudatus* | KF218730 | KF218659 | KF218712 | Ungurului cave, Şuncuiuş, Pădurea Craiului, ROM; NA902 |  |
| *Niphargus laticaudatus* | KF218722 | KF218658 | KF218687 | Grueţ cave, Roşia, Pădurea Craiului, ROM; NA905 |  |
| *Niphargus laticaudatus* | KF218717 | KF218660 | KF218699 | Corbasca cave, Sighiştel, ROM; NA909 |  |
| *Niphargus transsylvanicus* | KF218733 | - | KF218715 | Osoi cave, Vârciorog, ROM; NA904 |  |
| *Niphargus andropus* | KF218725 | KF218655 | - | Măgura cave , Sighiştel, ROM; NA942 |  |
| *Niphargus sp.*3 | KF218719 | KF218719 | KF218713 | Drăcoaia cave, Sighiştel, ROM; NA943 |  |
| *Niphargus sp.* 4 | KF218716 | KF218653 | KF218714 | Ciur Izbuc cave , Roşia, ROM; NA944 |  |
|  |  |  |  |  |  |

Sequences were compiled from the following studies:

Altermatt F., Alther R., Fišer C., Jokela J., Konec M. et al. (2014) Diversity and distribution of freshwater amphipod species in Switzerland (Crustacea: Amphipoda). Plos One 9(10): e110328. doi:10.1371/journal.pone.0110328

Esmaeili-Rineh S., Sari A., Delić T., Moškrič A., Fišer C. Molecular Phylogeny of the Subterranean Genus *Niphargus* (Crustacea: Amphipoda) in the Middle East: A Comparison with European Niphargids. Zoological Journal of the Linnean Society (in revision).

Fišer C., Sket B., Trontelj P. (2008) A phylogenetic perspective on 160 years of troubled taxonomy of Niphargus (Crustacea: Amphipoda). Zoologica Scripta 37:665-680.

Fišer C., Zagmajster M., Zakšek V. (2013) Coevolution of life history traits and morphology in female subterranean amphipods. Oikos 122: 770-778.

Flot J.F., Bauermeister J., Brad T., Hillebrand-Voiculescu A., Sarbu S.M., Dattagupta S. (2014) Niphargus–Thiothrix associations may be widespread in sulphidic groundwater ecosystems: evidence from southeastern Romania. Molecular Ecology 23: 1405-1417.

McInerney et al. (2014) The ancient Britons: groundwater fauna survived extreme climate change over tens of millions of years across NW Europe. Molecular Ecology 23: 1153-1166.

Meleg I.N., Zakšek V., Fišer C., Kelemen B.S., Moldovan O.T. (2013) Can environment predict cryptic diversity? The case of Niphargus inhabiting Western Carpathian groundwater. Plos One 8(10), e76760. doi:10.1371/journal.pone.0076760.

Švara V., Delić T., Rada T., Fišer C. Molecular phylogeny of *Niphargus boskovici* (Crustacea: Amphipoda) reveals a new species from epikarst. (in revision).

Trontelj P., Douady C.J., Fišer C., Gibert J., Gorički Š., Lefébure T., Sket B., Zakšek V. (2009) A molecular test for cryptic diversity in groundwaters: how large are the ranges of macro-stygobionts? Freshwater Biology 54: 727-744.

Trontelj P., Blejec A., Fišer C. (2012) Ecomorphological convergence of cave communities. Evolution 66: 3852-3865.
