# Supplementary material for: Redescription of two subterranean amphipods Niphargus molnari Méhely, 1927 and Niphargus gebhardti Schellenberg, 1934 (Amphipoda, Niphargidae) and their phylogenetic position
Source: Zookeys. 2015 Jun 24;(509):53–85. doi: 10.3897/zookeys.509.9820 (PMC4493343; doi:10.3897/zookeys.509.9820)
Supplement: Supplementary material 3 — Original descriptions of Niphargus molnari and Niphargus gebhardti [file zookeys-509-053-s003.doc]

**Supplementary file 3.** Original descriptions of *N. molnari* and *N. gebhardti*

***Niphargus molnari* n. sp.**,

Méhely, L. 1927: Új férgek és rákok a magyar faunában. Neue Würmer und Krebse aus Ungarn. Budapest 1927, 1–19.

Language of description: Hungarian

Type locality of species: Mánfai-kőlyuk Cave

*”*The snow-white animal is approximately 10 mm long, male larger then female. Eyless, 1-4. pereion segments strecth deeper then 5.; lower side of 5.-7. pereion segments heart-shaped (figure 5.). Free ends of pleon segments are pointed (figure 6.). First article of the big antenna is as long as the second, the third article is 2/3 of the second; flagellum has 17 articles. Basal article of the accesory flagellum is half times longer then the neighbouring flagellum article; second accesory flagellum is rather small, 1/4 of the basal article. First article of the small antenna is tiny, the 2. and 3. articles are equally long. 6. article of the 1. and 2. gnathopod is helmet-shaped and much more wider then 5. article. 1. and 2. uropods are short, the 3. is rather long, especially of the males; basal article is short, 2. article of external shaft is 2/3 of the 1. Internal shaft is scraggy. Telson is short, deeply inclined, round-edged. This new amphipoda species is named after in honour of Mr István Molnár…” (Figure 1.)


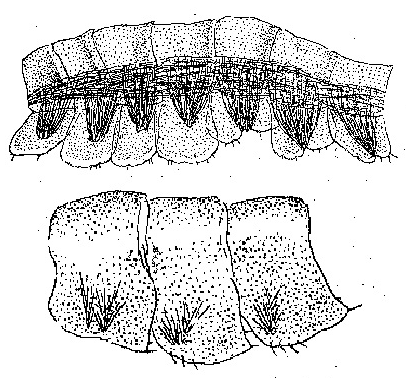


**Figure 1:** Drawings (pereionites and pleonites) from the original description of *N. molnari*.

***Niphargus foreli gebhardti* n. subsp.**

Schellenberg, A. (1934) Amphipoden aus Quellen, Seen und Höhlen. *Zoologischer Anzeiger,* 106, 200–209.

Language of description: German

Type locality of species: Abaligeti Cave

„ Die Art stammt aus einem ganz anderen Biotop als die vorige, nämlich aus kleinen, von Tropwasser gespeisten Tümpeln der Höhle Abaliget im Mecsekgebirge, Ungarn (Gebhardt coll.). Es liegen mir vor: 1 juv. 4 mm, 1 ♀ mit halbentwickelten Oostegiten 7 mm und 1 ♂ 7 mm. Trotz des abweichenden Biotops sind die Unterschiede von *transsylvanicus* nicht bedeutend. Sie drücken sich vor allem in der Länge der I. Antenne aus, die nur, ähnlich wie bei *foreli* 1/3 so lang ist wie der Körper. Ferner ist das Telson (Abb. 3a) kaum länger als breit.

Von sonstigen Unterschieden seinen erwähnt: Die 7 gliedrige Geißel der II. Antenne ist so lang wie das IV. + ½ V. Stielglied. Palpus der I. Maxille mit 5 Borsten, die Innenlade des Maxillarfußes mit 2 glatten Endstacheln. Beide Zahlen mögen bei älteren Tieren höher sein. Metacarpus der Gnathopoden (Abb. 3b) etwas breiter als bei transsylvanicus. Dactylus des III. und IV. Pereiopoden ohne Innenstachel. Basis des V.-VII. Pereiopoden elliptisch, wie bei auerbachi, hinten distal rundlish ausgezogen. Hinterrand auch beim ♂ konvex. III. Uropod des ♂ = 1/3 Körperlänge.” (Figure 2.)

**
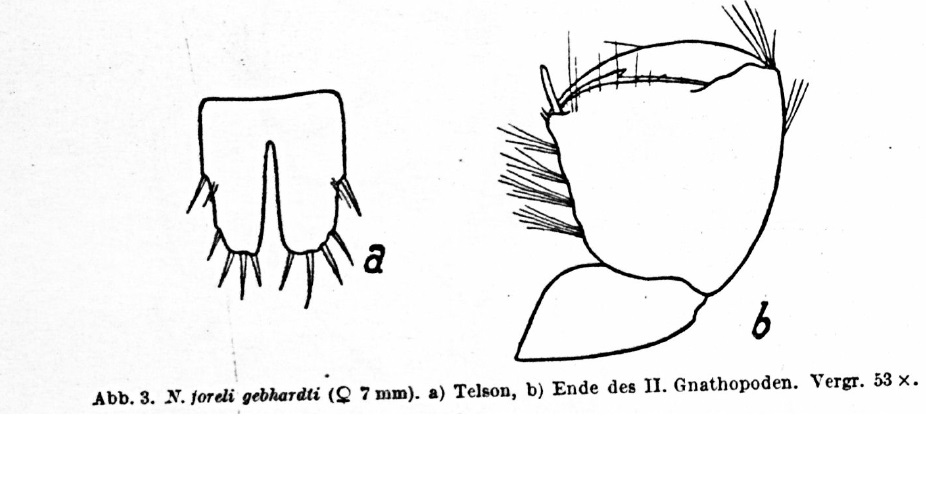
**

**Figure 2:** Drawings (telson and Gnathopod II propodus) from the original description of *N. foreli gebhardti*.
